# Real‐world treatment outcomes of medicines used in special situations (off‐label and compassionate use) in oncology and hematology: A retrospective study from a comprehensive cancer institution

**DOI:** 10.1002/cam4.6360

**Published:** 2023-07-26

**Authors:** Sandra Fontanals, Anna Esteve, Andrea González, Cristina Ibáñez, Javier Martínez, Ricard Mesía, Ana Clopés

**Affiliations:** ^1^ Pharmacy Department, Catalan Institute of Oncology (ICO)‐Hospitalet, Institut d’Investigació Biomèdica de Bellvitge (IDIBELL), School of Medicine Universitat de Barcelona (UB) Barcelona Spain; ^2^ Research Management Unit (Unitat de Gestió de la recerca: UGR), Medical Oncology Department, Catalan Institute of Oncology (ICO), Badalona‐Applied Research Group in Oncology (B‐ARGO) Germans Trias I Pujol Research Institute (IGTP) Badalona Spain; ^3^ Medical Oncology Department Catalan Institute of Oncology (ICO), Badalona‐Applied Research Group in Oncology (B‐ARGO) Germans Trias I Pujol Research Institute (IGTP) Badalona Spain; ^4^ Pharmacy Department, Catalan Institute of Oncology (ICO)‐Hospitalet School of Health Sciences, Blanquerna Ramon Llull University Barcelona Spain; ^5^ Pharmacy Department Catalan Institute of Oncology (ICO)‐Hospitalet Barcelona Spain; ^6^ Pharmacy Department, Catalan Institute of Oncology (ICO)‐Hospitalet, Institut d’Investigació Biomèdica de Bellvitge (IDIBELL) School of Health Sciences, Blanquerna Ramon Llull University Barcelona Spain

**Keywords:** cancer, compassionate use, off‐label use, survival, treatment outcomes

## Abstract

**Purpose:**

Medicines in special situations (MSS) refer to off‐label or to unlicensed drugs under investigation (compassionate use). Our objectives were to evaluate characteristics and to estimate overall survival (OS), event‐free survival (EFS), and the duration of treatment (DT) of MSS used for cancer treatment at a multicentre comprehensive cancer institution.

**Methods:**

Retrospective cohort study on adult cancer patients for whom an MSS treatment was requested (January 2011–December 2020). A descriptive analysis was performed and median OS and EFS and 95% confidence intervals (CIs) were estimated. Survival curves were stratified by type of tumor, ECOG (Eastern Cooperative Oncology Group) performance status (PS), age, sex, treatment stage and type of drug (mechanism of action and target).

**Results:**

Treatment was initiated in 2092 episodes (1930 patients) out of 2377 MSS episodes (2189 patients) requested, 33% for hematological treatment and 87% for advanced stage cancer. Median OS (months) was 21.1 (95% CI 19.4–22.7), median EFS was 5.6 (95% CI 5.1–6.0) months, and median DT was 4.5 [0.0; 115.3] months.

OS and EFS statistically significantly favored female patients, ECOG PS ≥2 episodes showed worse OS and EFS outcomes (*p* < 0.0001). Statistically significant differences in survival were found within solid and hematological cancer, disease stage, drug mechanism of action, and type of cancer (*p* < 0.001) but not for age. Survival outcomes by tumor subtype and drug are presented both globally and separately based on disease stage.

**Conclusion:**

MSS uses are practiced across almost all cancer types, mostly for advanced disease. ECOG PS ≥2, along with advanced disease, was related to worse survival. Information about real‐world outcomes is valuable and contributes to better decision‐making regarding MSS and our experience in this field could be of interest for other colleagues.

## Abstract 2

The study provides a real world data analysis of outcomes of off‐label and compassionate uses in a large cohort of oncology and hematology patients from a comprehensive and multicenter cancer institution. Main findings showed that survival outcomes differ between hematology and oncology, and a higher ECOG and advanced disease stage were associated with worse survival. Enhancing our understanding of real‐world outcomes associated with off‐label and compassionate offers valuable insights that can improve clinical decision‐making and contribute to the expanding body of knowledge in this field.

## 1 INTRODUCTION

Innovation and research in cancer are extremely active and the incorporation of new medicinal products into the therapeutic armamentarium implies a challenging, cumbersome, and long process at a regulatory level for the drug to be marketed and used in clinical practice.^1^ Briefly, all medicinal products need to be granted a marketing authorization by a regulatory body (European Medicines Agency [EMA] in European Union [EU], Food and Drug Administration [FDA] for United States of America [USA]). Afterwards, each member state decides about its own marketing reimbursement conditions for the drug within its National Health Service.^2^, ^3^ Since this process may take even longer than 18 months, the patient need for drug use may not be synchronized with the regulatory timeline.

Off‐label use (OLU) generally refers to the use of drugs outside their indication, as approved by regulatory agencies, and are common in cancer care.^3^ For some EU member states, OLU includes also compassionate use (CU) of investigational drugs regarding drugs that are being scientifically studied but are yet to be approved by the licensing authorities for clinical practice and are available through individual patient use request or expanded access programs. Nowadays, there is a lack of worldwide consensus on OLU definition and legislation. Indeed, even differences in labelling exist between USA and EU.^4^, ^5^ Moreover, in the EU, each member state has its own regulations for OLU prescribing and its use is frequently regulated by reimbursement restrictions since it is an economic challenge for healthcare systems.^6^

In 2009, Spain adopted a specific legislation ruled by the national Royal Decree No. 1015/2009^7^ that regulates the use of Medicinal products in special situations (MSS), applied to three scenarios: OLU, CU, and the use of drugs licensed in foreign countries but not authorized in Spain (Foreign drugs use). Health system is decentralized, and the budget is managed at a regional level. Thus, in Catalonia, a Spanish region with a population of 7.5 million (5.5 million adults) in 2022, the Catalan Health Service establishes specific drug use policies also affecting MSS use.^8^

According to the literature, oncology/hematology, psychiatry, and rheumatology are clinical areas of interest regarding OLU.^3^, ^9^, ^10^, ^11^, ^12^ Based on an EMA report, 13%–71% of adult patients with cancer will receive at least one chemotherapy as OLU during their course of treatment.^3^, ^13^ However, data on OLU (or on MSS use as a wider concept) health outcomes in real‐word practice in oncology are scarce and heterogenic, coming from small studies with wide variability in their design, sample size and pathologies involved, being extremely difficult to compare and extrapolate their results.^3^, ^4^, ^6^, ^9^, ^12^, ^13^, ^14^, ^15^, ^16^, ^17^ Moreover, few studies have evaluated the clinical outcomes of OLU of medicines in cancer treatment in terms of effectiveness, mostly based instead on cost and reimbursement issues.^6^, ^10^, ^12^, ^18^, ^19^, ^20^, ^21^, ^22^, ^23^, ^24^, ^25^

The Catalan Institute of Oncology (ICO) is a comprehensive multicentre cancer institution providing clinical oncology‐hematology care for approximately 45% of adult cancer patients in Catalonia. In 2022, ICO provided tertiary level cancer coverage for an adult population of 3,203,000 inhabitants. Regarding MSS use, to provide equity within patients, criteria for MSS eligibility are agreed and all MSS requests are reviewed by a multidisciplinary and multicentre group delegated from the ICO Pharmacy and Therapeutics committee (P&T), focusing on the evidence supporting MSS and weighting the potential benefits and risks of its use.

According to our institution MSS approved procedures, requesting physicians have to submit an MSS application form that includes clinical rationale and supporting evidence. This form has evolved through time to add relevant data such as patient performance, expected treatment benefit (according to ESMO Magnitude of Clinical Benefit Scale Score (ESMO‐MCBS v1.1),^26^ or expected overall response rate according to clinical trial. A favorable geriatric evaluation is required to accept a request for patients aged ≥75 years. Efficacy, safety, and cost are discussed individually by the committee in scheduled meetings, based on the clinical evidence and consideration of the available alternatives (including clinical trials). Safety and survival outcomes are collected subsequently.

In the present study, we retrospectively describe the cohort characteristics and report the results of real‐world MSS data with the aim to determine the overall survival (OS) of patients for whom an approved MSS was initiated from 2011 to 2020 in our institution, globally and separately for hematology (HEM) and oncology (ONC) population as valuable information in cancer setting. We also sought to assess event‐free survival (EFS), duration of MSS treatment (DT), and the reasons for discontinuation of MSS treatments.

## 2 METHODS

We conducted a retrospective multicentre cohort study on adult cancer patients. Eligibility criteria were patients aged ≥16 years old, treated at one of 3 ICO centres; ICO Girona, ICO Badalona, or ICO Hospitalet (Catalonia, Spain), and for those whom an MSS use for cancer treatment was requested from January 2011 to December 2020.

MSS requests for supportive care or unique dose treatment were excluded from the study. Patients could be eligible for MSS use more than once during their treatment. Each request was considered as an “episode”.

Eligible patients were identified through the institutional clinical record and cancer treatment database, which is periodically cross‐referenced with the public mortality register allowing to determine the mortality status of study subjects (up to June 2022). A relational database was created for the study, according to the law of Personal Data Protection and Digital Rights guarantee (EU 2016/679). Patients were followed up until April 8th, 2022.

Regarding descriptive variables, the retrieved information from each episode of MSS included: patient birth date, sex (female/male), tumor localization, date of diagnose, treatment context (first line or induction treatment versus refractory or relapsed disease for HAEM episodes; (neo)adjuvant, localized stage, advanced or metastatic disease for ONC episodes) and ECOG performance status (PS) at MSS episode initiation (0–4).

Cancer treatment information collected included: the number of treatments previous to MSS (excluding radiotherapy, surgery, or treatments not administered or dispensed at the Spanish hospital facilities such as hormonotherapy [aromatase inhibitors, fulvestrant or exemestan]); the types of agents used as MSS (drugs were identified by their International non‐proprietary name) and their mechanisms of action: targeted oral agents (TT), monoclonal antibodies (mAb), chemotherapy (CHT), hormone derivative therapy (HT), immunomodulatory agent, or other); the drug molecular target (for oral TT and mAb), monotherapy or combination treatment, and route of administration (oral, intravenous, subcutaneous or other); the MSS Initiation date; and the MSS stopping date for treatments discontinued within the study period.

Reason for MSS treatment discontinuation was registered (toxicity, progression, patient's decision, treatment completion, alternative treatment initiation, or other reasons). The adverse events (AE) related to treatment discontinuation due to toxicity were thoroughly reviewed. For those MSS eventually not administered, the reason for not starting MSS was recorded.

The study main endpoint was OS, defined as the time (in months) from the initiation of MSS until the date of death from any cause, lost to follow‐up, or administrative censoring, whichever occurred first. As a secondary endpoint, EFS was defined as the time (in months) from the date of MSS initiation until the date of disease progression, discontinuation of treatment, death from any cause, lost to follow‐up, or administrative censoring, whichever occurred first. DT was defined as the time (in months) from the date of MSS initiation until the date of the last administered dose of MSS (for discontinued treatment at the closing study date), the date of death from any cause, lost to follow‐up, or administrative censoring, whichever occurred first.

DT, EFS, and OS were also analyzed according to ECOG PS (0–1 vs. ≥2), sex (female/male), age at the time of MSS request (<75 vs. ≥75 years), treatment context, and mechanism of action of MSS drug.

As an exploratory survival analysis, OS and EFS were analyzed according to tumor type (within the five most frequent HEM and ONC tumors) and according to molecular target (for oral TT and mAb requested frequently [more than 25 times]).

### 2.1 Statistical analysis

A descriptive analysis of the study was performed, based on demographic, treatment, and pathology characteristics extracted from the patient's chemotherapy and medical records.

Characteristics were described using medians and interquartile ranges for continuous variables and frequencies and percentages for categorical variables. Median OS and median EFS and 95% Confidence Intervals (CIs) were estimated with the Kaplan–Meier (KM) method, and survival curves were stratified by type of tumor, ECOG PS, age, sex, treatment stage and type of drug (mechanism of action and target) and were compared with the log‐rank test. All statistical analyses were performed with the R v. 4.1.2 statistical software.

## 3 RESULTS

From January 2011 to December 2020, 46,749 patients received cancer treatment across the three centers included in the study: 80% of them for solid neoplasm treatment.

**FIGURE 1:**
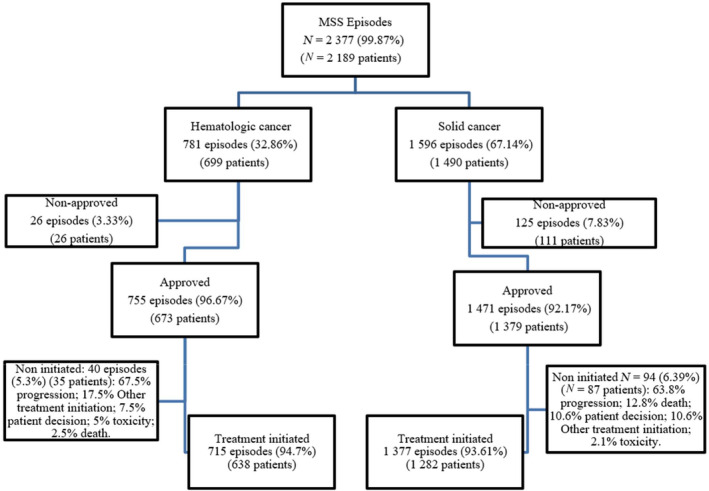
Flow chart.

Our search yielded 2189 patients that met inclusion criteria. The percentage of MSS requests for cancer treatment accounted for 5% of patients treated with systemic treatments at the 3 ICO sites. One third of the MSS requests were for hematological malignancies and two‐thirds for solid neoplasms. MSS episodes per patient ranged between 1 and 4, although most patients (92.5%) had a unique episode of MSS use. Most MSS requests were approved but for 6% of episodes (*n* = 151) (3.3% of HAEM and 7.8% of ONC requests), the committee denied MSS treatment. Within the approved treatments, 5.3% of hematologic cancer treatments (*n* = 40) and 6.4% of solid tumor treatments (*n* = 94) were not eventually initiated, most of them due to rapid disease progression. Reasons for the exclusion and detailed numbers are shown in the patients' flow‐chart (Figure 1).

Here, we present the analysis of 2092 episodes of MSS, corresponding to 1930 patients for whom an MSS was initiated within the study period. Median age at MSS initiation was 61.3 years (range: 17.1–92.2), 17.4% of episodes corresponded to patients aged ≥75 years old and half of the treatments were for women patients. Sixteen percent of ONC and 30% of HEM requests were for patients with an ECOG PS of 2 or higher (for ONC, 3% and for HEM 1.5% had an ECOG PS >2). Thirty‐eight percent of the episodes had received at least two previous lines of cancer treatment before MSS initiation but 45% had not received a previous systemic treatment line. It must be acknowledged that previous interventions such as radiotherapy, surgery, or hormonal treatment for breast or prostate cancer (aromatase inhibitors, exemestane, or fulvestrant) were not counted as previous lines of treatment as they are not administered nor dispensed at the Spanish hospital facilities and not registered in the treatment application.

Characteristics of initiated MSS episodes are presented in Table 1.

**TABLE 1:** Characteristics of patients initiating treatment with an MSS.

	Total			Hematological cancer			Solid tumor		
	Global, *N* = 2092	Early disease, *N* = 278	Advanced disease, *N* = 1814	Global, *N* = 715	Induction/debut^a^, *N* = 121	R/R, *N* = 594	Global, *N* = 1377	Early stage, *N* = 157	A/M, *N* = 1220
Gender, *n* (%):									
Female	1051 (50.2%)	155 (55.8%)	896 (49.4%)	315 (44.1%)	51 (42.1%)	264 (44.4%)	736 (53.4%)	104 (66.2%)	632 (51.8%)
Male	1041 (49.8%)	123 (44.2%)	918 (50.6%)	400 (55.9%)	70 (57.9%)	330 (55.6%)	641 (46.6%)	53 (33.8%)	588 (48.2%)
Median age [Min‐Max], years (y)									
mAge at diagn. (y)	58.2 [1.0; 89.4]	57.5 [17.0; 85.2]	58.3 [1.0; 89.4]	56.4 [17.0; 87.7]	60.7 [17.0; 85.2]	55.7 [17.5; 87.7]	59.0 [1.0; 89.4]	54.4 [20.3; 83.9]	59.5 [0.98; 89.4]
mAge at MSSi (y)	61.3 [17.1; 92.2]	58.7 [17.1; 85.8]	61.5 [18.8; 92.2]	60.0 [17.1; 92.2]	63.2 [17.1; 85.8]	59.4 [18.8; 92.2]	61.8 [19.7; 89.8]	55.8 [25.3; 84.5]	62.4 [19.7; 89.8]
Age at start of MSS, *n* (%)									
<75 y	1727 (82.6%)	229 (82.4%)	1498 (82.6%)	597 (83.5%)	91 (75.2%)	506 (85.2%)	1130 (82.1%)	138 (87.9%)	992 (81.3%)
≥75 y	365 (17.4%)	49 (17.6%)	316 (17.4%)	118 (16.5%)	30 (24.8%)	88 (14.8%)	247 (17.9%)	19 (12.1%)	228 (18.7%)
ECOG PS, *n* (%):									
0	330 (15.8%)	87 (31.3%)	243 (13.4%)	68 (9.5%)	19 (15.7%)	49 (8.25%)	262 (19.0%)	68 (43.3%)	194 (15.9%)
1	1327 (63.4%)	160 (57.6%)	1167 (64.3%)	434 (60.7%)	74 (61.2%)	360 (60.6%)	893 (64.9%)	86 (54.8%)	807 (66.1%)
≥2	435 (20.8%)	31 (11.2%)	404 (22.3%)	213 (29.8%)	28 (23.1%)	185 (31.1%)	222 (16.1%)	3 (1.91%)	219 (18.0%)
Number of chemotherapy lines prior to request, *n* (%):									
0	944 (45.1%)	153 (55.0%)	783 (43.2%)	204 (28.5%)	90 (74.3%)	114 (19.2%)	740 (53.7%)	71 (45.2%)	669 (54.8%)
1	360 (17.2%)	89 (32.0%)	271 (14.9%)	209 (29.2%)	31 (25.6%)^b^	178 (30.0%)	151 (11.0%)	58 (36.9%)	93 (7.62%)
≥2	788 (37.7%)	36 (12.9%)	760 (41.9%)	302 (42,2%)	–	302 (50.8%)	486 (35.3%)	28 (17.8%)	458 (37.5%)

Abbreviations: MSS, medicine in special situation; *N*, number of episodes; y, years; mAge, median age [Min‐Max] mAge at diagn., median age [Min‐Max] at diagnosis; mAge at MSSi, median age [Min‐Max] at MSS initiation. R/R, refractory/relapsed; for oncology: A/M, advanced/metastatic; Early disease, early stage for oncology treatments+ induction/debut treatment for hematology; Early stage, (neo)adjuvant or localized stage.

^a^ For hematology: Induction: induction to remission treatment (debut of disease, first line of treatment).

^b^ Previous treatments correspond to treatment of previous hematological disease (but first treatment for disease for whom an MSS was requested) or reinduction treatments.

Globally, 268 different drug schemes were requested and administered to the 1930 patients included for 51 different onco‐hematological diseases. MSS requests were mostly for refractory/relapsed (R/R) hematologic malignancies (83%) or advanced/metastatic (A/M) solid tumor stages (86%). Overall, drugs were administered as monotherapy in two‐thirds of episodes and oral exclusive administration accounted for 40% of episodes. Seventy‐four percent of the overall requests as MSS (1542 requests: 1141 for oncology and 401 requests for hematology) involved drugs and indications supported by stronger evidence, which were eventually approved by the EMA and the Spanish Medicines Agency, with a positive reimbursement decision within Spain up to May 2023. The remaining 550 requests were OL uses of drugs not reviewed by medicines agencies (518 requests) or through compassionate‐expanded access programs (32 requests) of drugs that eventually did not have regulatory approval or/and reimbursement positive decision.

In our study, there were 261 early‐stage requests (for neo/adjuvant, localized disease in oncology or first‐line for hematology). For 194 of them (67 for hematology and 127 for oncology), stronger evidence supported the drugs, leading to their eventual approval by the EMA. These drugs were used in anticipation, considering that the new drug would offer better results than the standard treatment. However, for 30% of the requests (84), the reasons for their use varied, including foreign drugs, contraindication of the use of standard treatment, or evidence based on clinical trials or cooperative group protocols without interest from pharmaceutical companies to pursue indication review by the EMA.

According to their mechanism of action, the most frequently administered were mAbs (37.5%) and oral targeted therapies (35.7%), either as a unique agent or in combination, whereas CHT accounted for 17.9% of global MSS episodes. While in HEM, the rate of mAb use almost doubled the use of oral TT, for ONC, proportion of use was similar for both families. To gain specific knowledge about the drugs involved in these categories, a subclassification of drugs according to their specific molecular target was performed. As a result, most frequently used drugs for ONC were: immune checkpoint inhibitors targeting programmed death ligand L1/1 (antiPD‐L1/PD‐1, *n* = 346), anti‐vascular endothelial growth factor therapy (anti‐VEGF, *n* = 156), epidermal growth factor receptor tyrosine kinase inhibitors (IK‐EGFR, *n* = 99), poly adenosine diphosphate‐ribose polymerase) inhibitors (IPARP, *n* = 104), and human epidermal growth factor receptor (HER2) inhibitors (anti‐HER2 *n* = 77); for HCL, anti‐CD20 mAbs (*n* = 126), anti‐CD30 mAbs (*n* = 70), global Janus kinase inhibitors (iJAK, *n* = 46), and Bruton Kinase inhibitors (iBK, *n* = 36). Detailed information about the number of episodes by tumor type and drugs used by cancer types for HEM and ONC population can be found in Tables S1–S3 in Supplementary Materials, respectively.

### 3.1 Study endpoints

Median OS for the study global population (*n* = 2092) was 21.1 (95% CI 19.4–22.7) months (Figure S1). Median EFS was 5.6 (95% CI 5.1–6.0) months. Median DT was 4.5 months (0.0; 115.3) and MSS treatment lasted more than 3 months in two‐thirds of episodes (*n* = 1369). Patients were followed up during a median of 17.4 months. At the end of the study period, 91% of treatments had been discontinued mainly due to disease progression (65.6%), followed by defined treatment completion (19%) and toxicity (13.7%).

Discontinuation of treatment due to hematologic, gastrointestinal, neurological, liver, and renal events were the most frequent. AE related to treatment discontinuation, along with their corresponding toxicity grades (when available in clinical records), and AE classification by drug are presented in Tables S4–S6 in Supplementary Materials.

Outcomes in episodes initiating MSS for global population as well as separately for hematological cancer and solid tumor are reported in Table 2.

**TABLE 2:** Outcomes in episodes initiating MSS.

	Total, *N* = 2092	Hematological cancer, *N* = 715	Solid tumor, *N* = 1377
Overall survival (months), median (95% CI)	21.1 (19.4–22.7)	34.2 (26.6–40.2)	18.1 (16.6–20)
Event free‐survival (months), median (95% CI)	5.6 (5.1–6)	4.9 (4.2–5.6)	5.8 (5.5–6.4)
MSS treatment duration (months), median [Min‐Max]	4.5 [0.0; 115.3]	3.0 [0.0; 103.3]	5.3 [0.0; 115.3]
MSS treatment duration ≥3 months, *n* (%)	1369 (65.4%)	456 (63.8%)	913 (66.3%)
Discontinued treatment at the end of the study, *n* (%)	1904 (91.0%)	644 (90.1%)	1260 (91.5%)
Reason for MSS discontinuation, *n* (%)			
Progression	1249 (65.6%)	334 (51.8%)	916 (72.7%)
Restricted treatment duration	362 (19.0%)	194 (30.1%)	168 (13.3%)
Toxicity	261 (13.7%)	107 (16.6%)	154 (12.2%)
Other reasons	32 (1.7%)	9 (1.4%)	22 (1.7%)
Follow‐up time (months), median [Min‐Max]	17.4 [<0.1; 129.9]	21.2 [0.1; 129.9]	16.2 [<0.1; 129.8]
Patients alive at the end of the study, *n* (%)	701 (33.5%)	290 (40.6%)	411 (29.8%)

Abbreviations: CI, confidence interval; MSS, medicines in special situations; *n*, number.

Survival analysis of global population according to ECOG PS, sex, age at initiation of treatment, drug mechanism of action, and cancer stage retrieved interesting results. Figures are shown in Supplementary Materials (Figures S2–S6). Episodes related to an ECOG PS of 2 or higher showed worse OS outcomes (months) (ECOG 0: 41.6; ECOG 1: 23.6; ECOG ≥2: 5.9; *p* < 0.0001) and EFS (months) (ECOG 0: 11.6; ECOG 1: 6.4; ECOG ≥2: 2; *p* < 0.0001). Regarding sex, OS, and EFS statistically significantly favored female (OS: 25.5 versus 17.1 months [*p* < 0.001]; EFS: 6.6 versus 4.6 months; [*p* < 0.001]). Statistically significant differences in survival were also found within the type of drug (TT, mAb, CHT, HT, or other, *p* < 0.001). However, no survival differences were detected within episodes of patients aged more than 75 years over younger patients.

**FIGURE 2:**
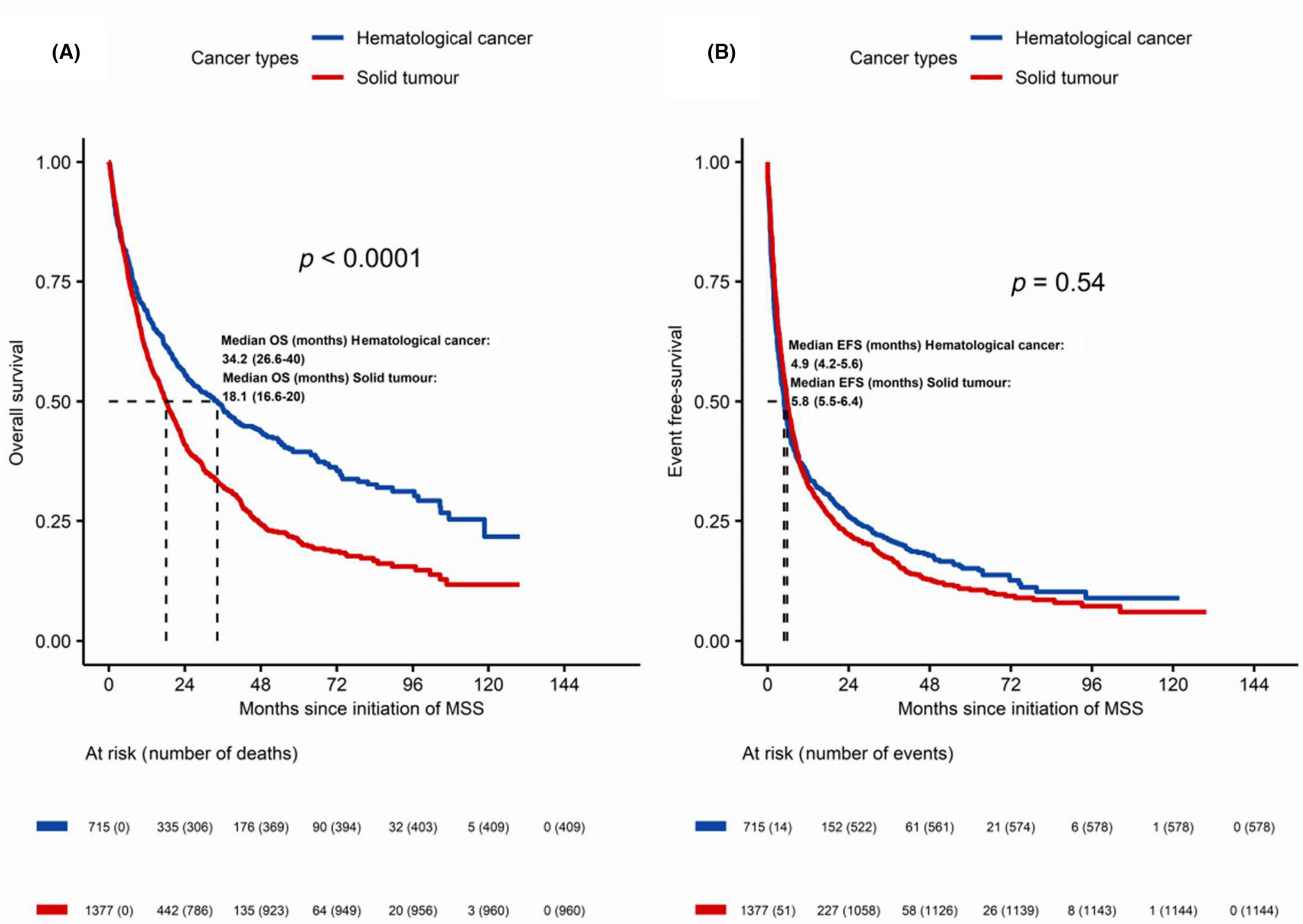
Overall survival (OS) (A) and event‐free survival (EFS) (B) according to cancer type.

As for type of neoplasm, median OS was statistically significantly different for HEM versus ONC episodes (34.2 vs. 18.1 months; *p* < 0.0001), whereas no significant differences were found in EFS (4.9 vs. 5.8 months). Results are shown in Figure 2. Median DT was 3.0 months for HEM episodes and 5.3 months for ONC. Both for HEM and ONC episodes, OS and EFS analysis according to ECOG PS showed statistically significant differences for ECOG 0–1 over ECOG ≥2 episodes (months) (HEM cancer: ECOG 0: 53.7; ECOG 1: 48.7; ECOG ≥2: 8.6; *p* < 0.0001); ONC: ECOG 0: 36.3; ECOG 1: 18.7; ECOG ≥2: 4.4; *p* < 0.0001). With respect to sex according to tumor type, statistically significant survival differences were only observed within ONC episodes (23.3 vs. 13.8 months; *p* < 0.0001).

As far as the treatment setting is concerned, for HEM episodes, median OS and EFS for induction/initial stage treatment were better than for refractory or resistant disease (OS: 53.7 vs. 31.9 months; *p* = 0.078). However, the differences were statistically significant only for EFS (7.7 vs. 4.7 months; *p* < 0.001). For ONC episodes, survival results clearly favored localized disease over advanced/metastatic treatment setting (OS: 42.1 vs. 14.3 months; *p* < 0.0001; EFS: 16.8 vs. 5.1 months); *p* < 0.0001).

We also explored whether the type of drug was potentially related to longer survival outcomes. In our study, although OS and EFS statistically differed by the mechanism of action (*p* < 0.001) both for HEM and ONC episodes, different patterns were observed. Among HEM, the longest median OS was observed with immunomodulator treatment (65.3 months), followed by mAb treatment (45.3 months) and oral TT (34.2 months). For solid cancer episodes, TT showed similar OS results to HT (21.0 vs. 20.9 months).

Results from survival analysis for global population and separately for HEM and ONC episodes are shown in Tables 3 (OS) and 4 (EFS).

**TABLE 3:** Overall survival analysis according to patient, drug, and disease characteristics.

		Global population, (*n* = 2092)			Hematological cancer, *N* = 715			Solid cancer, *N* = 1377		
		*n*	Median OS in months (95% CI)	*p*	*n*	Median OS in months (95% CI)	*p*	*n*	Median OS in months (95% CI)	*p*
Sex	Female	1051	25.5 (22.9–30)	0.0001	315	37.9 (26.9–56.9)	0.22	736	23.3 (20.4–26.1)	<0.0001
	Male	1041	17.1 (15.1–19.5)		400	29 (23.6–40)		641	13.8 (11.7–15.8)	
Age at start of MSS	<75 y	1727	20 (18.4–22)	0.56	597	34.9(26.6–46.1)	0.2	1130	17.3 (15.8–18.9)	0.78
	≥75 y	365	23.3 (21.6–28.4		118	32.7 (21.8–53.7)		247	22.7 (20.8–25.9)	
ECOG PS	0	330	41.6 (34.6–48.8)	0.0001	68	53.7 (40–68.2)	<0.0001	262	36.3 (32.1–45.9)	<0.0001
	1	1327	23.6 (21.6–26)		432	48.7 (37.6–65.3)		393	18.7 (17.1–21.1)	
	≥2	435	5.9 (4.7–7)		213	8.6 (6.6–12.3)		222	4.4 (3.7–5.6)	
Drug family	mAb (iv)	785	22.6 (18.7–28.4)	0.0001	287	45.3 (34–63.8)	<0.0001	498	17.3 (13.9–20)	0.01
	TT (or)	746	22.5 (19.7–25)		169	34.2 (23.1–53.7)		577	21 (17.9–23.6)	
	CHT	86	13.8 (11.8–18.1)		175	17.6 (13.4–24)		199	12 (9–17.2)	
	HT	86	20.9 (13.8–23)		–	–		86	20.9 (13.8–23)	
	Immunomodulator	54	65.3 (34.9‐NA)		53	65.3 (34.9‐NR)		–	–	
	Others	47	29.1 (16.2‐NA)		31	21.5 (16.1‐NR)		16	NR (8.8‐NR)	
Disease stage					121	Induction/initial stage: 53.7 (26.6‐NR)	0.08	22	Neoadjuvant setting NR (NR‐NR)	<0.0001
								105	Adjuvant setting: NR (NR‐NR)	
					594	Relapsed/refractory disease: 31.9 (24.9–38.8)		30	Localized disease: 42.1 (23.1‐NR)	
								1220	Advanced/metastatic disease: 14.3 (13.1–16.3)	

Abbreviations: CHT, chemotherapy; CI, confidence interval; HT, hormone‐therapy; iv, intravenous, mAb, monoclonal antibody; MSS, medicines in special situations; *N*, number; NR, not‐reached; or, oral; OS, overall survival; TT, targeted therapy; y, years.

**TABLE 4:** Event free‐survival analysis according to patient, drug, and disease characteristics.

		Global population			Hematologic cancer			Solid cancer		
		*n*	Median EFS in months (95% CI)	*p*	*n*	Median EFS in months (95% CI)	*p*	*n*	Median EFS in months (95% CI)	*p*
Sex	Female	1051	6.6 (5.8–7.4)	0.0001	315	5.1 (4–6.4)	0.92	736	7.4 (6.4–8.6)	<0.0001
	Male	1041	4.6 (4.1–5.3)		400	4.9 (3.9–5.8)		641	4.6 (3.9–5.4)	
Age at start of MSS	<75 y	1727	5.3 (4.9–5.8)	0.1	597	4.8 (3.9–5.3)	0.1	1130	5.7 (5.3–6.2)	0.1
	≥75 y	365	7.4 (5.7–9.9)		118	7.1 (5–14.4)		247	7.4 (5.6–10.2)	
ECOG PS	0	330	11.6 (9.3–16.1)	0.0001	68	13.1 (7.6–30.9)	<0.0001	262	11.6 (9.3–16.1)	<0.0001
	1	1327	6.4 (5.8–7.1)		432	6.6 (5.4–8.9)		1371	6.7 (6–7.4)	
	≥2	435	2 (1.8–2.5)		201	2.4 (1.9–3.1)		193	2.5 (1.9–2.9)	
Drug family	mAb (iv)	785	5.1 (4.5–6)	<0.0001	287	4.5 (3.7–5.2)	<0.0001	498	5.8 (4.7–6.9)	0.01
	TT (or)	746	7.6 (6.6–8.9)		169	11.1 (5.8–20.4)		577	7.2 (6.3–8.5)	
	CHT	86	3.5 (2.9–3.9)		175	3.3 (2.8–4.4)		199	3.5 (2.8–4.2)	
	HT	86	7.6 (4.6–11.3)		–	–		86	7.6 (4.6–11.3)	
	Immuno modulator	54	11.7 (7.7–24.6)		53	14.1 (7.7–24.6)		–	–	
	Others	47	5.8 (4.1–12.3)		31	4.2 (1.9–12.3)		16	10 (4.8‐NR)	
Disease stage					121	Induction/initial stage: 7.7 (5.1–19.2)	0.08	22	Neoadjuvant setting: 5.1 (3.3‐NR)	<0.0001
								105	Adjuvant setting: NR (NR‐NR)	
					594	Relapsed/refractory disease 4.7 (3.9–5.3)		30	Localized disease: 16.8 (6.2‐NA)	
								1220	Advanced/metastatic disease: 5.1 (4.6–5.6)	

Abbreviations: CHT, chemotherapy; CI, confidence interval; EFS, event‐free survival; HT, hormone‐therapy; m, months; mAb, monoclonal antibody; MSS, medicines in special situations; *N*, number; NR, not‐reached; TT, targeted therapy; y, years.

Most frequent hematological malignancies were non‐Hodgkin (*n* = 199) and Hodgkin lymphoma (*n* = 57), acute myeloid leukemia (AML) (*n* = 86), myelodysplastic syndromes (MDS) (*n* = 79) and multiple myeloma (*n* = 47). Median OS observed for those pathologies ranged from the lowest 10.0 months for AML to the highest 65.2 months for MDS.

Most frequent solid neoplasms were thoracic (*n* = 361), genitourinary (*n* = 211), breast (*n* = 227), skin (*n* = 137), and gynecological (*n* = 133). Skin cancer obtained the longest median OS, 31.4 months, and genitourinary the shortest, 11.2 months. To provide information on the most frequent tumor types, we conducted a survival outcomes analysis, revealing variations in survival outcomes amongst the top‐five cancers for which MSS use was most frequently requested. Median OS, median EFS, as well as 12‐months and 36‐months survival rates for the most frequent tumors are available in Table S7 and Figure S7 in Supplementary Materials.

Given that the prognosis can vary depending on the tumor subtype and context, we conducted a survival analysis for each tumor subtype to describe the results within the included tumors. The corresponding analysis for hematology and oncology can be found in Tables S8 and S9 in Supplementary Materials.

Survival analysis according to molecular targets, within the oral TT and mAbs agents requested more than 25 times, showed that anti‐CD20 mAbs and Bcr‐Abl tyrosine kinase inhibitors were related to the best OS results in HEM (69.3 and 72.9 months, respectively), while anti‐HER2, PARP inhibitors, and ALK‐kinase inhibitors had the longest survival in ONC (36.2, 34.0, and 32.1 months, respectively). Survival data according to drug subclassification is available in Table S10 and details on frequency of mAb and TT according to subclassification for HEM and ONC are shown in Tables S11 and S12, respectively, in Supplementary Materials.

Interestingly, in 211 episodes (5%) from the global population, MSS treatment was maintained for more than 2 years, and in 34 (1.6%) patients, treatment lasted for more than 5 years. Within these long responders, no clear patient or cancer characteristic was identified except for the type of drug, which was commonly oral TT and mAb.

Finally, in the Supplementary Materials, specifically Tables S13 and S14, we provide a comprehensive description of survival results for drugs and indications that were eventually approved by regulatory agencies and requested at least 10 times. Furthermore, for informative purposes, we have included information from pivotal studies for those indications in columns juxtaposed with the study results. For oncology drugs, the score from the ESMO benefit clinical scale is also recorded. It is important to note that the characteristics of individual requests may vary slightly from the approved indication, and the study's design does not aim to compare results on a case‐by‐case basis.

## 4 DISCUSSION

Few data can be found in the literature regarding MSS survival outcomes in real world practice in oncology, mostly focused on one specific drug or pathology or with a small sample size.^18^, ^19^, ^20^, ^21^, ^22^, ^23^, ^24^, ^25^, ^27^, ^28^ To our understanding, this is the first large study to provide real‐world results in a cohort of patients treated with MSS for cancer in a multicentric comprehensive cancer institution, reporting overall and EFS and analyzing factors associated with survival outcomes. The proportion of patients for whom an MSS was requested MSS over the global number of patients treated at ICO during the study period was 5%, which is smaller than reported (17%–71%).^3^, ^11^ However, it must be acknowledged that in our study supportive care MSS were excluded. Additionally, there are other factors that influence that finding. First, as stated before, at ICO, the procedure and analysis regarding the use of MSS depends on the P&T Committee. According to its procedures, clinicians must complete an MSS form to justify their patient's treatment request and, if applicable, their alternative available treatments (including clinical trials), which must be supported by the head of their medical department to proceed to committee evaluation. A hospital pharmacist elaborates a supporting evidence review report. Requests are then discussed collegiately by a multidisciplinary and multicentre group to eventually accept or deny them. This procedure implies an accurate selection of requests.^29^ Besides, MSS frequently requested are prioritized for P&T evaluation, allowing to discuss and to agree criteria for their use, resigning from the need for a per‐patient basis request and eventually minimizing bureaucratic procedures regarding their use once approved. Moreover, ICO develops Clinical Practice Guidelines (ICOPraxis) for cancer treatment.^30^ During the review and elaboration process of a cancer treatment guideline, if efficacy and safety data support the inclusion of MSS drug use in institutional guidelines, it may be discussed and approved by the P&T Committee, also preventing from the individual basis request requirement. These circumstances may have influenced the low incidence of MSS found in our study with respect to EMA report.^3^

On the other hand, our findings suggest that MSS use is commonly practiced across most cancer types.

Over the last few years, the EMA approval process timelines have been lengthy. Furthermore, once medicines are granted marketing authorization, it is at the discretion of the company holding the authorization to determine in which EU countries the medicine will be marketed and when they will apply for pricing and reimbursement. This, along with the subsequent state reimbursement process, results in additional months before the drug becomes available on the market.^31^, ^32^ Consequently, this situation leads to an increasing number of requests for drugs pending price and funding decisions in our country, affecting MSS requests.

In our study, most episodes were for relapsed and advanced disease (87%). It is well‐known that metastatic cancer patients, who have exhausted previous standard lines of treatment are most likely to receive drugs used as MSS. Despite metastatic cancers being, in general, incurable, as cancer armamentarium and knowledge evolve, patients may keep a good functional status following disease progression on approved treatment regimens and be eligible to receive an MSS drug.^9^, ^11^, ^13^ However, in our study, even in patients with advanced stage disease, the level of prior treatment was not extensive, since only 38% of episodes received at least two previous lines of treatment, despite some differences amongst hematology and oncology episodes. It is important to highlight that previous interventions such as radiotherapy, surgery, or hormonal treatment (aromatase inhibitors) for cancer were considered as previous treatments since they were not included in the hospital treatment application records.

Nonetheless, this finding may reflect how new treatments strategies development, mainly driven by new molecular targets, have been focused on early lines of treatment, aiming to change expected cancer survival.

Consequently, novel drugs with positive results were used in anticipation as MSS, considering that the new drug would offer better results than the standard treatment.

During the 10‐year period of the study, cancer treatments have evolved considerably, with the emergence of targeted therapies and immunotherapy that has contributed to increased survival expectancy of many of the tumors evaluated. While traditional intravenous chemotherapy use has diminished, oral cancer treatments have increased explosively during the last decade. However, little data can be found on the literature about the prevalence of targeted therapy as OLU since these studies are not easy to perform because of this rapid evolution.^13^

Our main goal was to describe our cohort and to estimate survival outcomes of cancer therapies used as MSS in a real‐world setting. It must be considered that evidence compelling MSS may widely range from the use of therapies with evidence of strong benefits used in anticipation of their approval by regulatory agencies to the opposite of requests based on less or weak evidence of treatment benefits or that will neither be reviewed nor approved at a regulatory level.^13^, ^14^, ^17^ While our study found that 75% of requests were for drugs that were eventually approved by the EMA, which provides a stronger rationale for their use, it should be acknowledged that the clinical or disease characteristics of the requests may slightly differ from the marketed label. Thus, clinical results expected with MSS in a real‐world data setting may be extremely variable.

Survival analysis in our cohort showed interesting findings. ECOG PS 2 was statistically significant for OS and EFS over ECOG ≤1, thus patient PS could be considered as a predictor of potential effectiveness of an MSS. Arroyo et al. found comparable results regarding EPS and short treatment duration in their study, in which 168 cancer patients treated with MSS were analyzed. However, the survival results obtained in their study were less favorable.^33^

Differences in OS were also observed between sexes but only for solid cancers, with more favorable outcomes observed in women. Although there is recognized sexual dimorphism in the immune system response, the impact of patients' sex on the outcomes of cancer treatments remains poorly understood cancers.^34^, ^35^ We hypothesize that this finding in oncology may be associated with the tumor's location, which exhibits different patterns according to sex for certain solid tumors, in contrast to hematology. Regarding age, in our study, no differences in survival were observed, which, to our understanding, reinforces the need for applying patient eligibility criteria to select patients that are in good condition to receive MSS treatment despite their age, as considered in ICO MSS procedures.

In our study, the reason for MSS treatment discontinuation was toxicity in 13.7% of patients, which correlates with well‐known adverse safety profile of cancer drugs with hematological and gastrointestinal as most frequent AE causing treatment discontinuation. The duration of MSS treatment was short, less than 5 months, even shorter for hematological malignancies. However, a wide range of DT was observed. It must be highlighted that for one third of episodes treatment lasted less than 3 months, doubtingly obtaining a favorable benefit of treatment. On the other side, while it is difficult to conclude from this study that these therapies provided long‐term benefit, we found few episodes with prolonged DT, since for 5% of episodes treatment lasted more than 2 years and for 1.6% of episodes it lasted more than 5 years.

In our study, differences in survival according to the mechanism of action of MSS were also found. While better results in terms of OS for oral TT and mAbs could be expected due to their specific mechanism of action, interestingly, HT for ONC and immunomodulator agents for HEM showed improved survival results.

In order to pr[object HTMLSpanElement] 12(2):482. doi: 10.3390/cancers12020482.ovide a comprehensive understanding of the patient population and the tumor subtypes considered in our analysis, we provide data on survival outcomes according to tumor subtype. The pathologies for which MSS requests were more frequent were consistent with the prevalence of neoplasms and correlated with the pathologies that have been the focus of active research during the studied period.^36^, ^37^, ^38^, ^39^, ^40^, ^41^, ^42^, ^43^, ^44^, ^45^

Furthermore, for informative purposes, we have included survival information stratified by drug, juxtaposed with results from pivotal studies for the corresponding indications. Upon a simple numerical comparison, we found that the results obtained within our population for hormone therapy in prostate cancer and for ovarian (olaparib), lung (alectinib, lorlatinib, durvalumab), thyroid (lenvatinib), breast (palbociclib after aromatase inhibitors), head and neck (nivolumab), and melanoma (nivolumab, pembrolizumab, ipilimumab) were largely similar to the published results. However, it is important to note that for most hematological indications, data were lacking, and the results were not numerically comparable.

We acknowledge that our study has several limitations. First, it is an observational study prone to inherent limitations due to its retrospective nature. Second, a wide variety of cancers were included, some of which had limited representation, making it challenging to draw definitive conclusions. However, since we focused on describing the characteristics and outcomes of real‐world in patients for whom an MSS use for cancer treatment was requested, the actual distribution of cancer types was informative in itself.

Third, the study is limited by its reliance on data from three centers in a single institution, which may not be representative of treatment practices at other centers. Nonetheless, it should be noted that the ICO provided clinical oncology and hematology care for approximately half of the adult population in Catalonia.

Nevertheless, the main strength of our study lies in the assessment of survival and clinical outcomes in a cohort of more than 2000 patients with solid and hematological cancers, with a median follow‐up of 17.4 months over a 10‐year study period.

## 5 CONCLUSION

Information obtained from the present study is valuable and contributes to better decision‐making regarding MSS use. Our findings allow discussion within P&T Committee about clinical criteria that should be accomplished, previously to requesting an MSS use and reinforces the usefulness of MSS accurate discussion of requests to maximize chances of treatment benefit. The results obtained in our cohort helps us to increase knowledge about real world data results of treatments and, for the most frequent pathologies and drugs requested, allows us to benchmark them with those obtained in clinical trials and to increase knowledge about the drug. To conclude, we believe that our experience and procedures regarding OLU may be of interest to other colleagues and extrapolated to other countries.

## AUTHOR CONTRIBUTIONS

**Sandra Fontanals:** Conceptualization (equal); investigation (lead); project administration (lead); resources (supporting); supervision (lead); validation (lead); writing – original draft (lead). **Anna Esteve:** Conceptualization (equal); data curation (equal); formal analysis (lead); methodology (lead); validation (equal); visualization (supporting); writing – review and editing (equal). **Andrea González:** Data curation (equal); formal analysis (equal); methodology (supporting); validation (equal); visualization (equal). **Cristina Ibáñez:** Resources (supporting); validation (supporting); writing – review and editing (supporting). **Javier Martínez:** Resources (supporting); writing – review and editing (supporting). **Ricard Mesía:** Conceptualization (supporting); project administration (supporting); supervision (supporting); writing – review and editing (equal). **Ana Clopés:** Conceptualization (supporting); project administration (supporting); supervision (supporting); writing – review and editing (equal).

## FUNDING INFORMATION

No funding was received to assist with the study and the preparation of this manuscript.

## CONFLICT OF INTEREST STATEMENT

The author(s) declare(s) that there are no conflicts of interest regarding the publication of this article.

## ETHICS STATEMENT

The study protocol and statistical analysis plan were approved by the independent Ethics committee of the Bellvitge University Hospital (protocol code PR090/21‐UB110321) with a waiver of informed consent, due to its retrospective nature. The study was conducted in accordance with the Good Clinical Practice guidelines and the provisions of the Declaration of Helsinki.

## Supporting information


Figure S1.

Table S1.

## Data Availability

The datasets generated during and/or analyzed during the current study are not publicly available due to reasons of sensitivity but are available from the corresponding author on reasonable request.
