# Supplementary material for: Real‐world treatment outcomes of medicines used in special situations (off‐label and compassionate use) in oncology and hematology: A retrospective study from a comprehensive cancer institution
Source: Cancer Med. 2023 Jul 26;12(16):17112–25. doi: 10.1002/cam4.6360 (PMC10501253; doi:10.1002/cam4.6360)

SUPPLEMENTARY MATERIAL-FIGURES

Figure S1. A: Overall survival (OS) and B: Event Free-Survival (EFS) for overall population

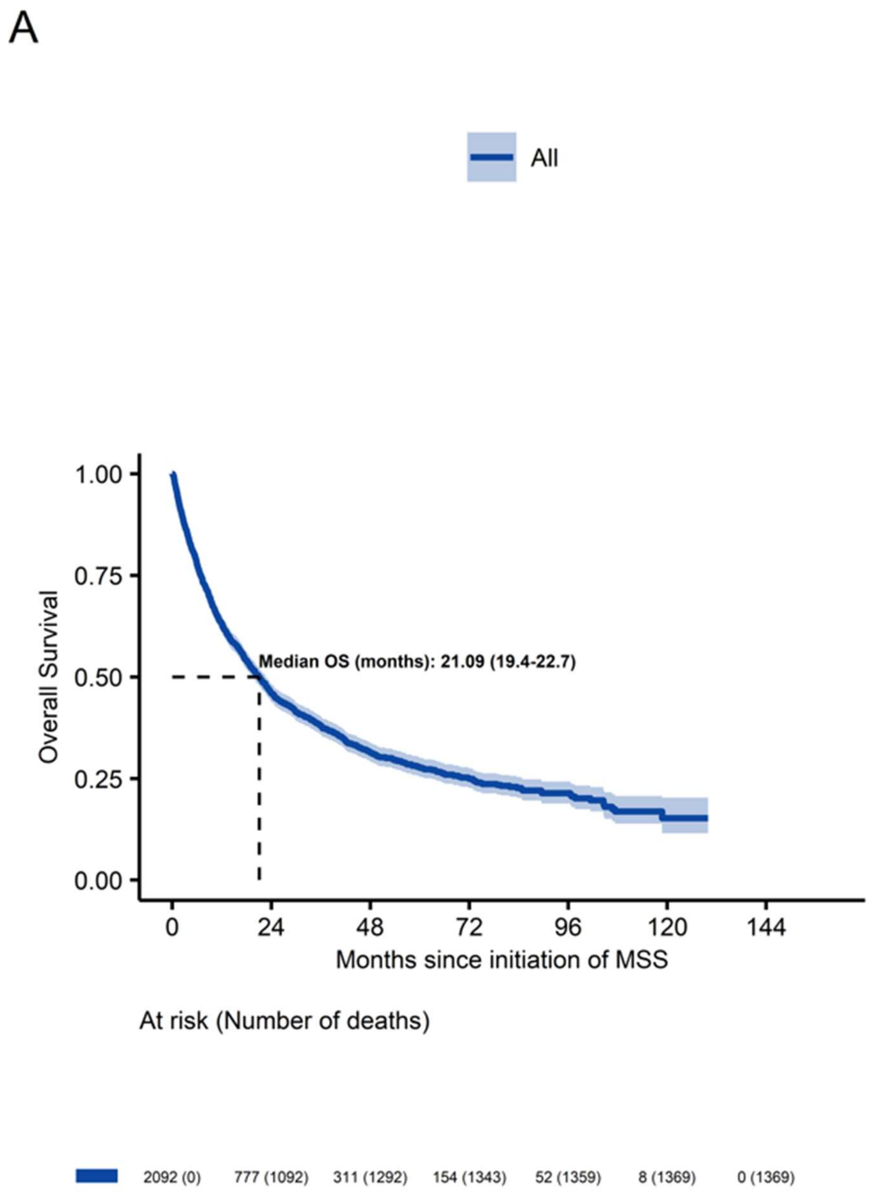

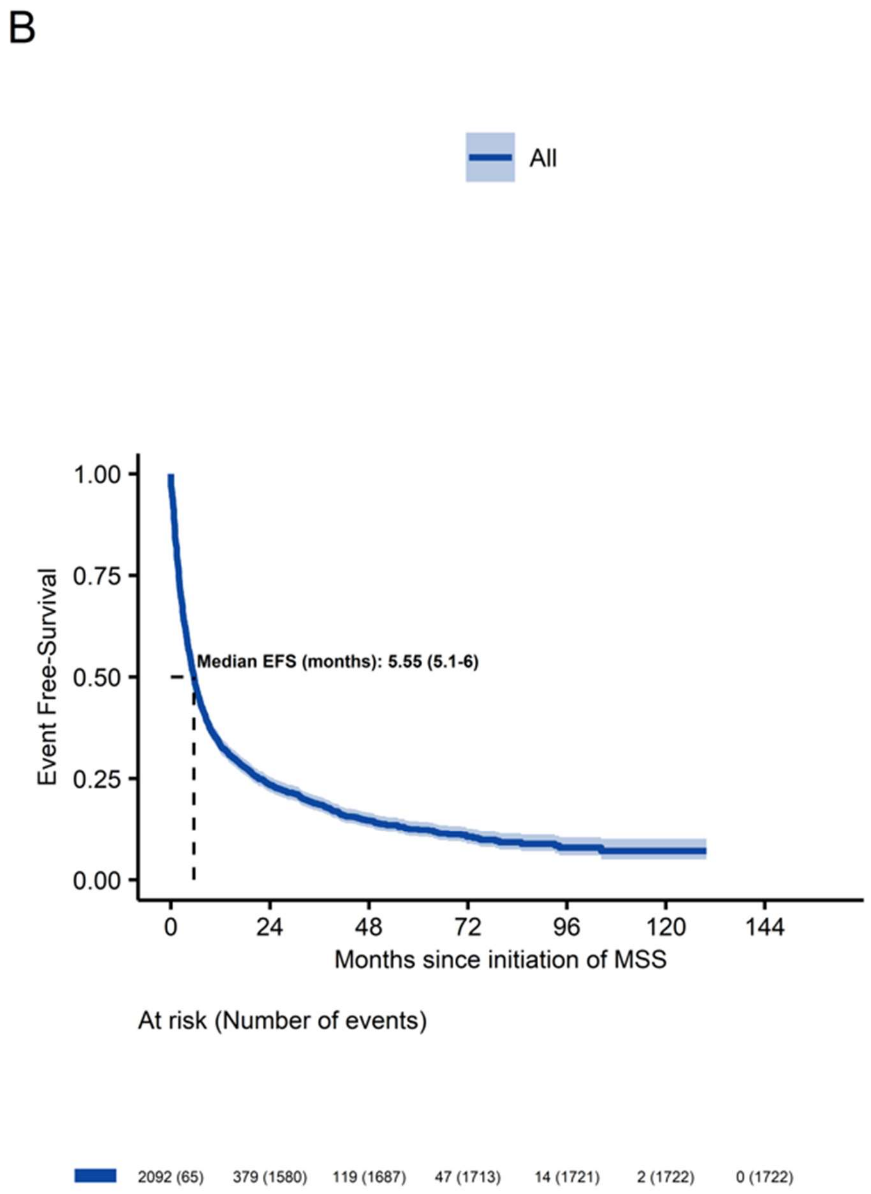

Figure S2. Overall survival according to ECOG PS for overall population (A), and for patients with hematologic cancer (B) and with solid tumour (C).

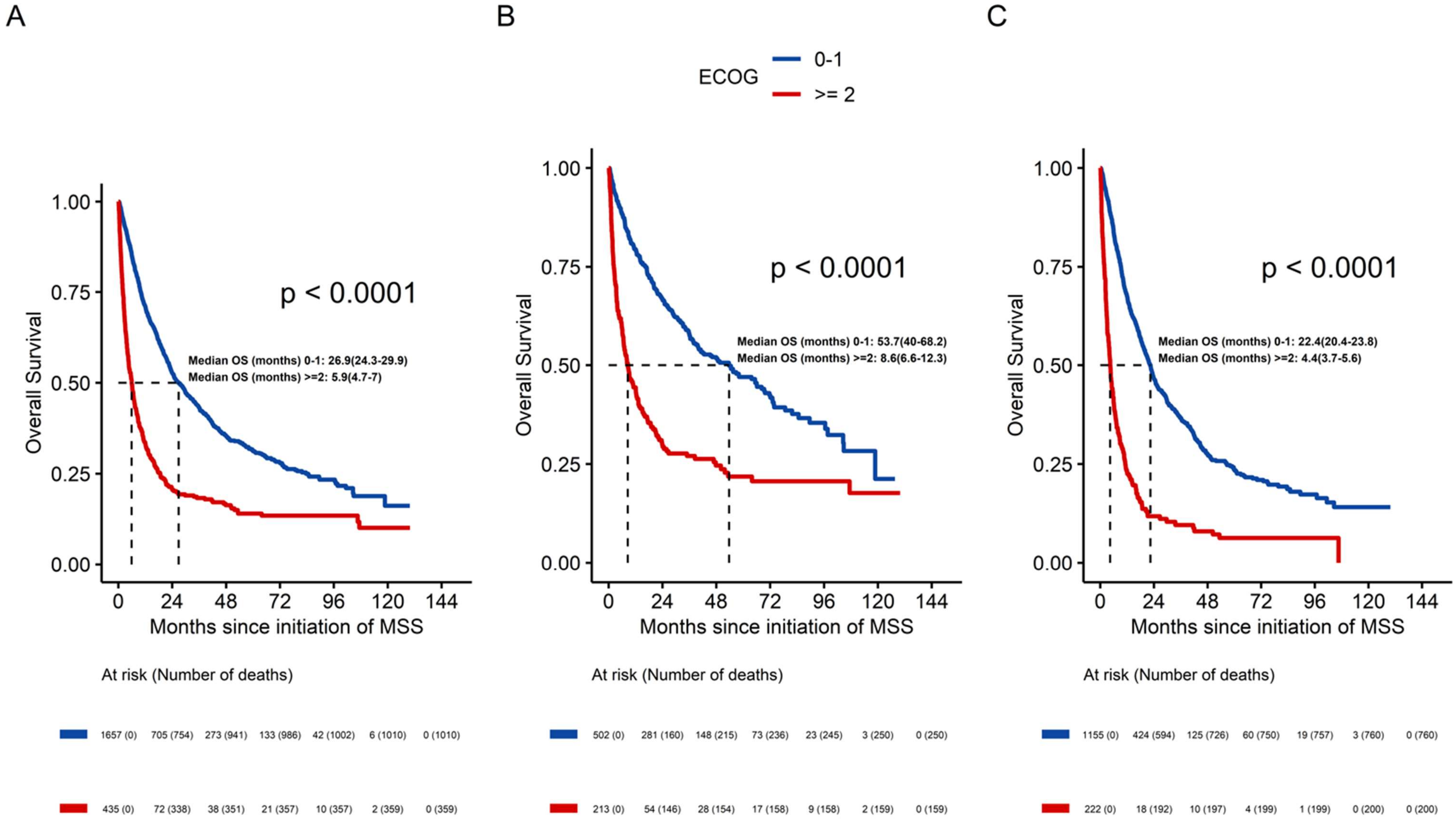

Figure S3. Overall survival according to sex for overall population (A), and for patients with hematological cancer (B) and with solid tumour (C).

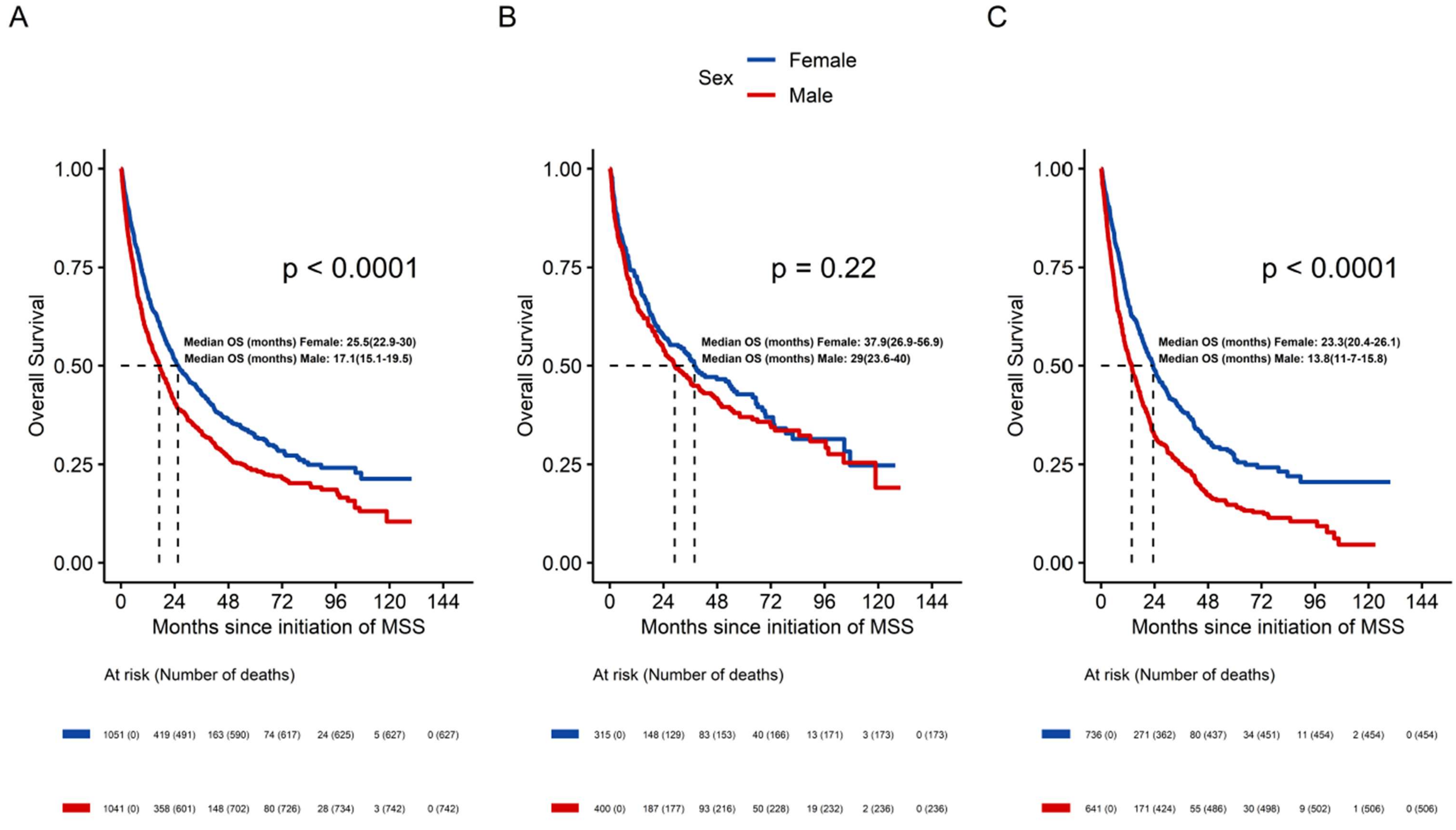

Figure S4. Overall survival according to age for overall population (A), and for patients with hematological cancer (B) and with solid tumour (C).

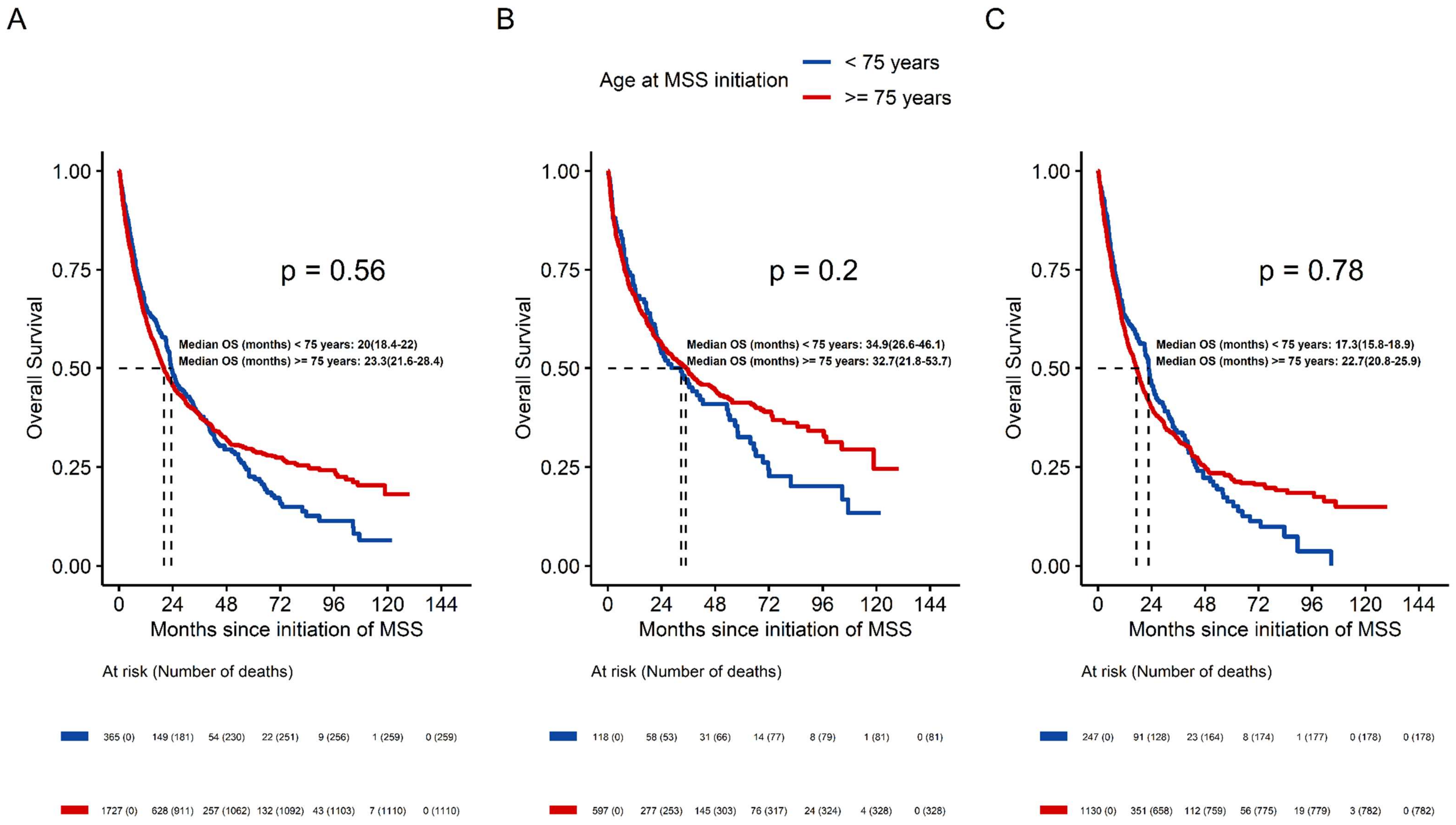

Figure S5. Overall survival according to disease stage for patients with hematological cancer (A) and for patients with solid tumour (B).

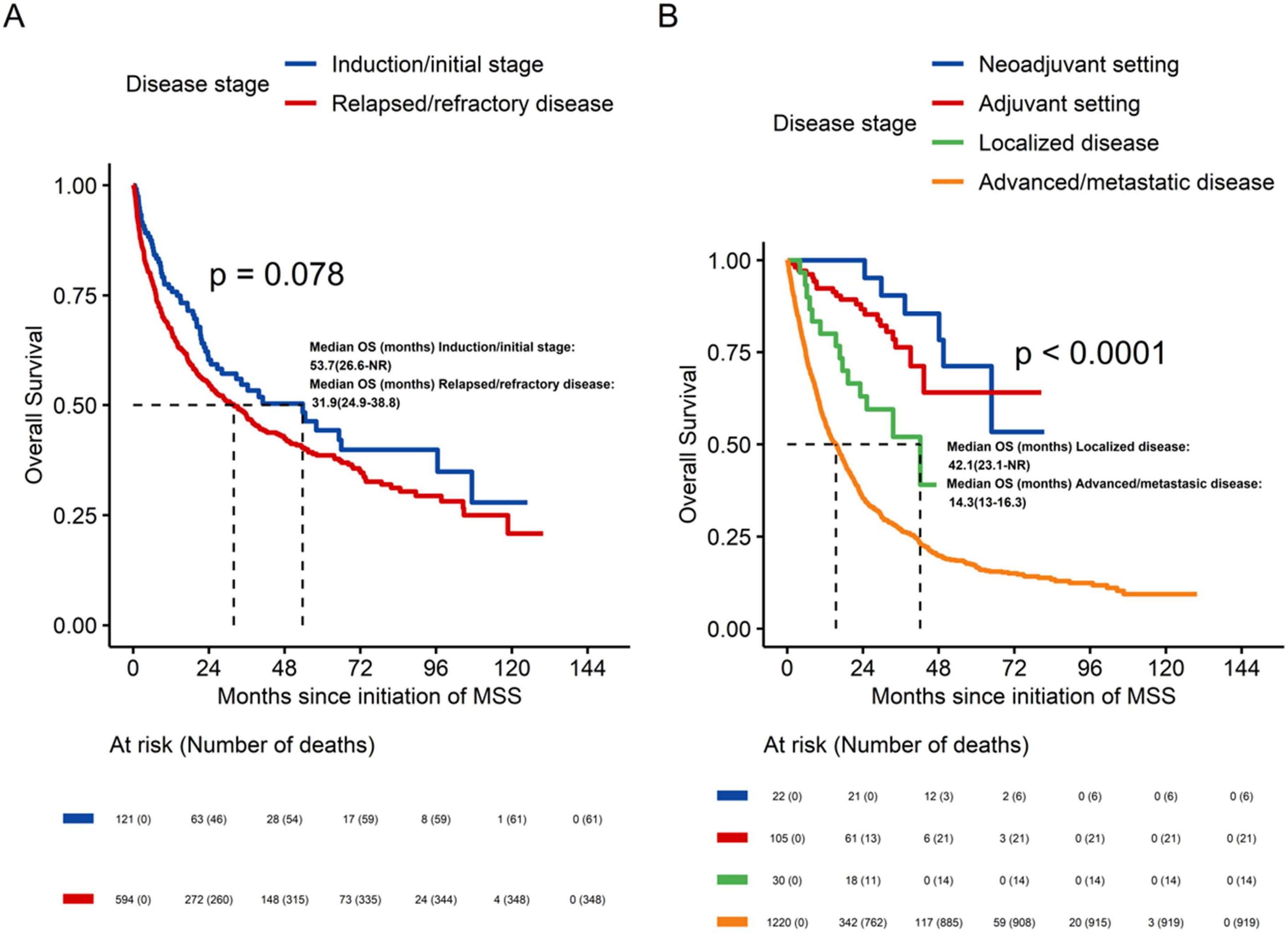

Figure S6. Overall survival according to MSS mechanism of action for global patients (A), patients with hematological cancer (B) and for patients with solid tumour (C).

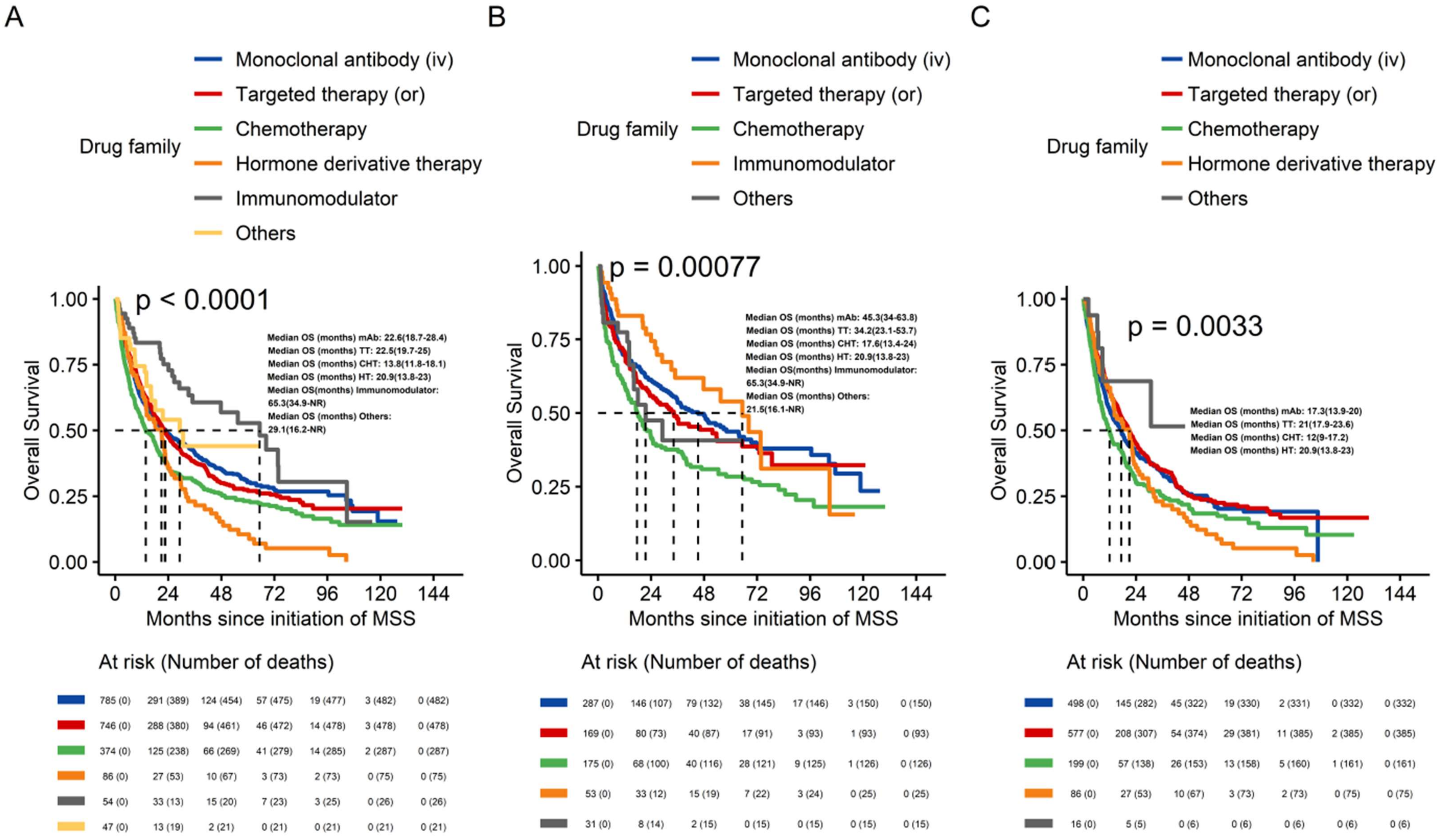

mAb: monoclonal antibody; TT: targeted therapy; HT: hormone-therapy; CHT: Chemotherapy.

Figure S7. Overall survival for most frequent tumours (hematological (A) and oncological (B) cancer)

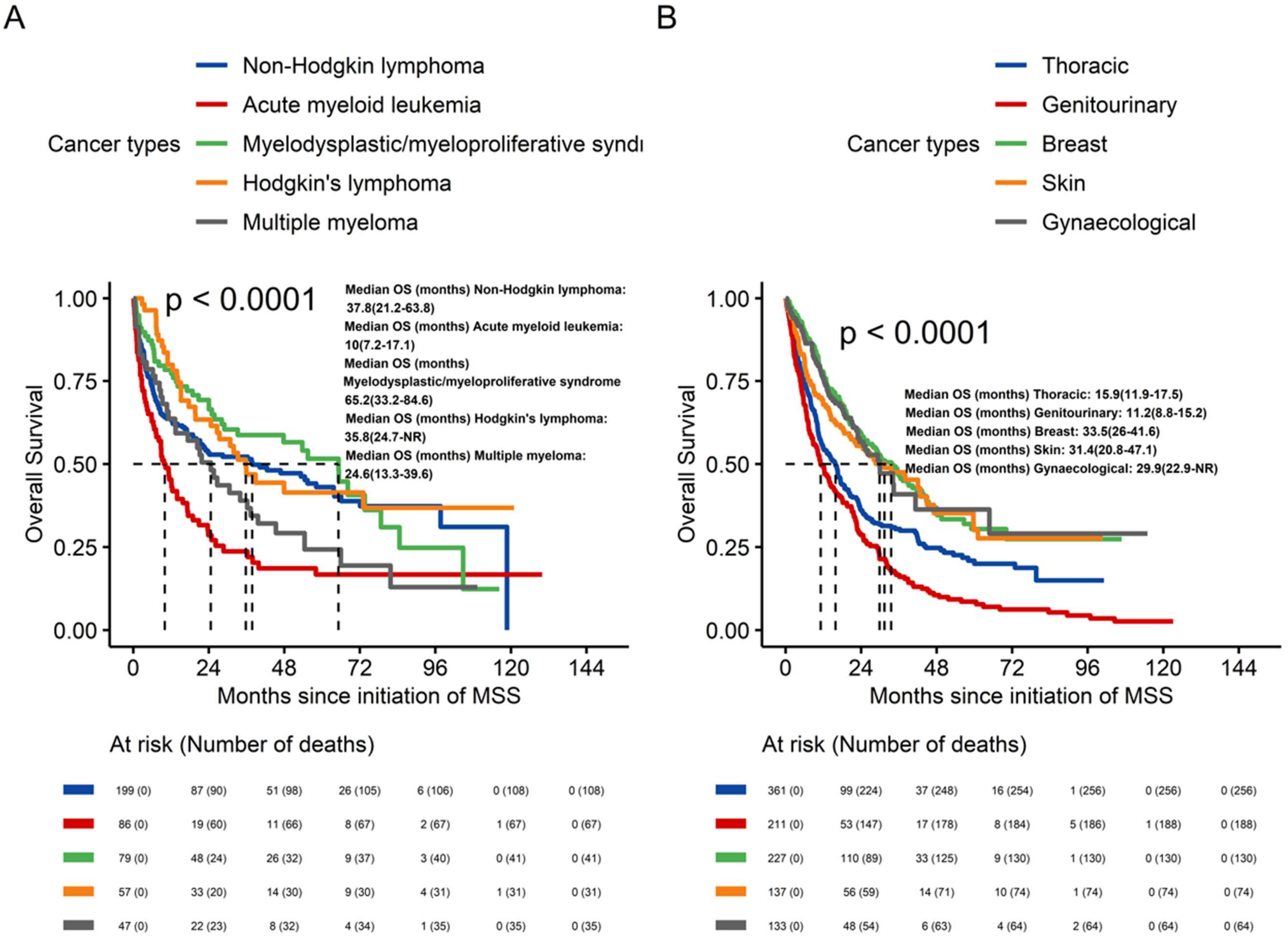

Supplement: Supplementary file 1 — Figure S1. [file CAM4-12-17112-s001.pdf]
