# Supplementary material for: Real‐world treatment outcomes of medicines used in special situations (off‐label and compassionate use) in oncology and hematology: A retrospective study from a comprehensive cancer institution
Source: Cancer Med. 2023 Jul 26;12(16):17112–25. doi: 10.1002/cam4.6360 (PMC10501253; doi:10.1002/cam4.6360)
Supplement: Supplementary file 2 — Table S1. [file CAM4-12-17112-s002.pdf]

SUPPLEMENTARY MATERIAL

Table S1. Number of episodes of Medication in Special Situations (MSS) by cancer types.

Hematological cancer N=715		Solid tumour N=1377	
**Cancer types**	**n (%)**	**Cancer types**	**n (%)**
Non-Hodgkin lymphoma	199 (27.8%)	Thoracic	361 (26.2%)
Acute myeloid leukaemia	86 (12.0%)	Genitourinary	211 (15.3%)
Myelodysplastic/myeloproliferative syndrome	79 (11.0%)	Breast	227 (16.5%)
Hodgkin's lymphoma	57 (8.0%)	Skin	137 (9.9%)
Multiple myeloma	47 (6.6%)	Gynaecological	133 (9.7%)
Acute lymphoblastic leukaemia	46 (6.4%)	Sarcoma	84 (6.1%)
Chronic lymphatic leukaemia	43 (6.0%)	Gastrointestinal	82 (6.0%)
Chronic myeloid leukaemia	43 (6.0%)	Head and neck	57 (4.1%)
Other hematological cancer[¥]	36 (5.0%)	Neuroendocrine	45 (3.3%)
Waldenström Macroglobulinemia	27 (3.8%)	Central nervous system	33 (2.4%)
Thrombocytopenic purpura	19 (2.7%)	Other solid tumour	7 (0.5%)
Cell aplasia	17 (2.4%)		
Amyloidosis	16 (2.2%)		

[¥]Other Hematological cancer includes requests (<5 each) for Hemolytic anaemia, Gammopathy, Histiocytosis, Hairy cell leukaemia, Prolymphocytic lymphoma and Mastocytosis.

Table S2. Drugs (used in monotherapy or in combinations) by cancer types (for drugs requested ≥ 25 times and cancer type frequency > 10%) for Hematological cancer.

Drug requested (n)	Cancer types	n (%)
Rituximab (n = 114)	Non-Hodgkin lymphoma	57 (50.00%)
	Thrombocytopenic purpura	14 (12.28%)
	Waldenström Macroglobulinemia	13 (11.40%)
	Other hematological cancer	13 (11.40%)
Brentuximab vedotin (n = 70)	Non-Hodgkin lymphoma	38 (54.29%)
	Hodgkin's lymphoma	31 (44.29%)
Ruxolitinib (n = 46)	Myelodysplastic/myeloproliferative syndrome	28 (60.87%)
	Acute lymphoblastic leukaemia	6 (13.04%)
	Non-Hodgkin lymphoma	5 (10.87%)
Ibrutinib (n = 36)	Chronic lymphatic leukaemia	17 (47.22%)
	Non-Hodgkin lymphoma	13 (36.11%)
	Waldenström Macroglobulinemia	6 (16.67%)
Bortezomib (n = 34)	Non-Hodgkin lymphoma	17 (50.00%)
	Amyloidosis	8 (23.53%)
	Multiple myeloma	7 (20.59%)
Venetoclax (n = 33)	Acute myeloid leukaemia	25 (75.76%)
	Chronic lymphatic leukaemia	7 (21.21%)
Eltrombopag (n = 28)	Cell aplasia	14 (50.00%)
	Other hematological cancer	6 (21.43%)
	Myelodysplastic/myeloproliferative syndrome	5 (17.86%)
	Thrombocytopenic purpura	3 (10.71%)
Lenalidomide (n = 27)	Myelodysplastic/myeloproliferative syndrome	13 (48.15%)
	Multiple myeloma	7 (25.93%)
	Non-Hodgkin lymphoma	3 (11.11%)

Table S3. Drugs (used in monotherapy or in combination) by cancer types (for drugs requested ≥ 25 times and cancer type frequency > 10%) for solid cancer.

Drug requested (n)	Cancer types	n (%)
Nivolumab (n = 142)	Thoracic	57 (40.14%)
	Skin	38 (26.76%)
	Genitourinary	22 (15.49%)
	Head and neck	20 (14.08%)
Pembrolizumab (n = 128)	Thoracic	78 (60.94%)
	Skin	28 (21.88%)
Osimertinib (n = 60)	Thoracic	59 (98.33%)
Olaparib (n=55)	Gynaecological	46 (80.7%)
	Breast	9 (15.79%)
Abiraterone (n = 55)	Genitourinary	55 (100%)
Palbociclib (n = 50)	Breast	48 (96%)
Niraparib (n = 45)	Gynaecological	45 (100%)
Dabrafenib (n = 41)	Skin	40 (97.56%)
Trastuzumab emtansine (n = 39)	Breast	37 (94.87%)
Carboplatin (n = 36)	Gynaecological	11 (30.56%)
	Thoracic	8 (22.22%)
	Breast	8 (22.22%)
	Genitourinary	5 (13.89%)
Atezolizumab (n = 35)	Genitourinary	21 (60%)
	Thoracic	6 (17.14%)
Bevacizumab (n = 34)	Central nervous system	15 (44.12%)
	Gynaecological	11 (32.35%)
Enzalutamide (n = 29)	Genitourinary	29 (100%)
Durvalumab (n = 28)	Thoracic	28 (100%)
Regorafenib (n = 28)	Gastrointestinal	14 (50%)
	Sarcoma	14 (50%)
Everolimus (n = 27)	Breast	12 (44.44%)
	Neuroendocrine	11 (40.74%)

Table S4. Adverse events causing treatment discontinuation.

Adverse events causing interruption of treatment	Number of times reported	%
	N=313 (261 patients)	
Hematologic event	58	18,53%
Gastrointestinal event	36	11,50%
Neurological event	30	9,58%
Liver impairment	29	9,27%
Renal impairment	28	8,95%
Dermatological affection	19	6,07%
Asthenia	17	5,43%
Infectious disease	17	5,43%
Lung impairment	17	5,43%
Cardiac impairment	13	4,15%
Circulatory	7	2,24%
Hypersensitivity-Allergy	6	1,92%
Pain	6	1,92%
Cardiac event	4	1,28%
Electrolytes alteration	3	0,96%
Mucositis	3	0,96%
Pulmonary embolism	3	0,96%
Hyperglycaemia	2	0,64%
Neurologic	2	0,64%
Hemorrhagic event	2	0,64%
Anorexia	1	0,32%
Arthralgia	1	0,32%
Arthritis	1	0,32%
Coagulation	1	0,32%
Cushing syndrome	1	0,32%
Fungal infection	1	0,32%
Hearing impairment	1	0,32%
Hypothyroidism	1	0,32%
Osteonecrosis	1	0,32%
Proctitis	1	0,32%
Psychotic attack	1	0,32%

Table S5. Type of adverse events by grade (as reported in clinical charts).

Grade of Adverse Event	Number of times reported
**AE Grade reported**	**119 (36%)**
**AE Grade not reported**	**194 (64%)**
**Grade 2**	**30 (9.6%)**
Asthenia	9
Circulatory: oedema	2
Dermatological: rash	1
Gastrointestinal: diarrhoea	8

Hematologic: anaemia	4
Liver impairment	1
Lung-pneumonitis	1
Mucositis	1
Neurological	3
Aphonia	1
**Grade 3**	**65 (20.8%)**
Asthenia	3
Cardiac	1
Circulatory: hypertension	1
Cushing syndrome	1
Dermatological	6
Gastrointestinal: diarrhoea, vomiting	15
Hematologic: neutropenia, anaemia	17
Liver impairment	11
Lung-pneumonitis	1
Mucositis	2
Neurological	3
Pain	3
Renal impairment	1
**Grade 4**	**22 (7.0%)**
Anorexia	1
Asthenia	1
Circulatory: arterial vasculopathy	1
Electrolytes alteration: hypokalaemia	1
Hematologic: neutropenia, thrombocytopenia	12
Liver impairment	2
Lung: pneumonitis	1
Neurologic: paresis	1
Renal impairment	2
**Grade 5**	**1 (0.3%)**
Renal impairment	1

Table S6. Frequency of drugs discontinued due to drug toxicities and related adverse events (AE).

**Drug and AE**	**Number of patients reporting AE; n=261 pts**
**Pembrolizumab (n=15)**	
Asthenia	2
Cardiac: Immune-mediated-heart-pericarditis	1
Dermatological: toxicoderma	1
Electrolytes alteration-hypokalaemia	1
Infectious disease	1
Liver impairment	2

Drug and AE	Number of patients reporting AE; n=261 pts
Lung impairment: pleural effusion	1
Lung: pneumonitis	2
Neurological- Immune-mediated	1
Immune-mediated-renal impairment	2
Renal impairment	1
**Nivolumab (n=16)**	
Asthenia	1
Cardiac impairment	1
Cardiac impairment-myocardiopathy	1
Dermatological condition	2
Immune-mediated gastrointestinal: diarrhoea	1
Immune-mediated-gastrointestinal: colitis	1
Hematologic: Hemolytic anaemia	1
Infectious disease	1
Liver impairment: Immune-mediated	2
Neurological: Immune-mediated paresis	1
Neurological: polymyalgia	1
Immune-mediated renal impairment	1
Renal impairment	2
**Regorafenib (n=10)**	
Asthenia	1
Circulatory: arterial vasculopathy	1
Gastrointestinal: diarrhoea	1
Gastrointestinal: gut perforation	1
Hematologic: anaemia	1
Liver impairment	5
**Brentuximab vedotin (n=9)**	
Hematologic: neutropenia	1
Hypersensitivity: allergy	1
Neurological: neuropathy	6
Renal impairment	1
**Rituximab (n=8)**	
Hematologic: febrile neutropenia	1
Hematologic: neutropenia	2
Hematologic: neutropenia, thrombocytopenia, anaemia	1
Mucositis	1
Neurological: encephalitis	1
Neurological: neuropathy	1
Pulmonary embolism	1
**Carboplatin (n=7)**	
Hematologic: febrile neutropenia	1
Hematologic: neutropenia	1
Hearing impairment	1
Hypersensitivity: allergy	2
Neurological: neuropathy	2

**Drug and AE**	**Number of patients reporting AE; n=261 pts**
**Eltrombopag (n=7)**	
Asthenia	1
Gastrointestinal: diarrhoea	1
Hyperglycaemia	1
Infectious disease	1
Liver impairment	1
Neurological: headache	1
Renal impairment	1
**Lenalidomide (n=7)**	
Asthenia	1
Gastrointestinal: diarrhoea	3
Hematologic: neutropenia, thrombocytopenia, anaemia	1
Hematologic: thrombocytopenia, anaemia	1
Infectious disease	1
**Niraparib (n=7)**	
Cardiac impairment	2
Circulatory: hypertension	1
Hematologic: anaemia	1
Hematologic: thrombocytopenia	2
Neurological: dizziness	1
**Alpelisib (n=6)**	
Dermatological: rash	2
Gastrointestinal: diarrhoea	1
Hyperglycaemia	1
Lung-pneumonitis	1
Osteonecrosis	1
**Bosutinib (n=6)**	
Circulatory: oedema	1
Gastrointestinal: diarrhoea	2
Hematologic: neutropenia, thrombocytopenia, anaemia	1
Liver impairment	1
Renal impairment	1
**Daratumumab (n=6)**	
Electrolytes alteration-hyperammonaemia	1
Gastrointestinal: colitis	1
Hematologic: neutropenia, thrombocytopenia, anaemia	1
Infectious disease	2
Renal impairment	1
**Ruxolitinib (n=6)**	
Circulatory: oedema	1
Dermatological: ulcer	1
Hematologic: thrombocytopenia	1
Hemorrhagic event: brain Hemorrhage	1
Infectious disease	1
Neurological: dizziness	1

Drug and AE	Number of patients reporting AE; n=261 pts
**Atezolizumab (n=5)**	
Infectious disease	1
Liver impairment: Immune-mediated	1
Lung: pneumonitis	1
Renal impairment	2
**Dabrafenib (n=5)**	5
Dermatological: rash	3
Hematologic: thrombocytopenia	1
Infectious disease	1
**Enzalutamide (n=5)**	
Asthenia	2
Neurological-restless legs syndrome	1
Pain	1
Psychotic attack	1
**Olaparib (n=5)**	5
Asthenia	1
Hematologic: thrombocytopenia, anaemia	2
Neurological: headache	1
Renal impairment	1
**Palbociclib (n=5)**	
Gastrointestinal: diarrhoea, anorexia	1
Hematologic: neutropenia	1
Hematologic: neutropenia, thrombocytopenia, anaemia	1
Renal impairment	2
**Ponatinib (n=5)**	
Cardiac impairment: stroke	1
Cardiac impairment: angina pectoris	1
Gastrointestinal: diarrhoea	1
Hematologic: neutropenia, thrombocytopenia, anaemia	1
Neurological: neuropathy	1
**Trastuzumab emtansine (n=5)**	
Cardiac impairment: myocardiopathy	1
Cardiac impairment: pericarditis	1
Hematologic: thrombocytopenia	2
Neurological: neuropathy	1
**Venetoclax (n=5)**	5
Hematologic: thrombocytopenia	1
Hematologic: neutropenia, thrombocytopenia, anaemia	1
Infectious disease	1
Liver impairment: cholestasis	1
Renal impairment	1
**Azacytidine (n=4)**	
Hematologic: neutropenia	2
Hematologic: neutropenia, thrombocytopenia, anaemia	1
Hematologic: thrombocytopenia	1

**Drug and AE**	**Number of patients reporting AE; n=261 pts**
**Bortezomib (n=4)**	
Hematologic: febrile neutropenia	1
Hematologic: thrombocytopenia	2
Neurological: neuropathy	1
**Durvalumab (n=4)**	
Lung-pneumonitis	3
Neurological: encephalitis	1
**Everolimus (n=4)**	
Gastrointestinal: diarrhoea	2
Hematologic: anaemia	1
Lung: pneumonitis	1
**Interpheron (n=4)**	
Asthenia	1
Dermatological	1
Hematologic: neutropenia, thrombocytopenia, anaemia	1
Hypersensitivity; allergy	1
**Afatinib (n=3)**	
Cardiac impairment: enzyme alteration	1
Gastrointestinal: diarrhoea	2
**Crizotinib (n=3)**	
Gastrointestinal: diarrhoea	1
Hematologic: neutropenia, thrombocytopenia, anaemia	1
Renal impairment	1
**Dasatinib (n=3)**	
Gastrointestinal: diarrhoea	1
Infectious disease	1
Lung impairment: pleural effusion	1
**Fluorouracil (n=3)**	
Gastrointestinal: vomiting	1
Hematologic: neutropenia	1
Mucositis	1
**Ibrutinib (n=3)**	
Dermatological condition: lumps	1
Infectious disease	2
**Idelalisib (n=3)**	
Gastrointestinal: diarrhoea, vomiting	1
Lung impairment	1
Lung impairment: bronchitis	1
**Lenvatinib (n=3)**	
Circulatory: hypertension	1
Coagulation disorder: bleeding	1
Electrolytes alteration: hypocalcaemia	1
**Nintedanib (n=3)**	
Asthenia	1
Gastrointestinal: diarrhoea	1

**Drug and AE**	**Number of patients reporting AE; n=261 pts**
Hematologic: neutropenia	1
**Midostaurin (n=3)**	
Dermatological	1
Gastrointestinal: nausea, vomiting	1
Liver impairment	1
**Sunitinib (n=3)**	
Cardiac impairment	1
Gastrointestinal: diarrhoea	1
Hypothyroidism	1
**Alectinib (n=2)**	2
Liver impairment	1
Renal impairment	1
**Bendamustine (n=2)**	
Hematologic: neutropenia, thrombocytopenia, anaemia	1
Neurological: delirium	1
**Bevacizumab (n=2)**	
Dermatological condition: fistula	1
Pulmonary embolism	1
**Blinatumomab (n=2)**	
Liver impairment	1
Neurological: aphasia	1
**Cabazitaxel (n=2)**	
Hematologic: thrombocytopenia, anaemia	1
Pain	1
**Cetuximab (n=2)**	
Hemorrhagic event: Hemoptysis	1
Liver impairment	1
**Clofarabin (n=2)**	
Hematologic: thrombocytopenia	1
Liver impairment: cholestasis	1
**Docetaxel (n=2)**	
Dermatological condition-rash	1
Neurological: neuropathy	1
**Gemtuzumab ozogamycin (n=2)**	
Liver impairment	2
**Imatinib (n=2)**	
Dermatological condition: fistula	1
Gastrointestinal: diarrhoea	1
**Ipilimumab (n=2)**	
Liver impairment	2
**Obinutuzumab (n=2)**	
Liver impairment	1
Neurological	1
**Sorafenib (n=2)**	
Dermatological condition: rash	1

**Drug and AE**	**Number of patients reporting AE; n=261 pts**
Liver impairment	1
**Trastuzumab (n=2)**	
Lung: pneumonitis	1
Renal impairment	1
**Vandetanib (n=2)**	
Proctitis	1
Renal impairment	1
**Abemaciclib:** Gastrointestinal: diarrhoea	1
**Abiraterone + prednisone:** Cardiac impairment: ischemia	1
**Asciminib:** Lung impairment: pleural effusion	1
**Axitinib:** Asthenia	1
**Brigatinib:** Lung: pneumonitis	1
**Cabozantinib:** Gastrointestinal: diarrhoea, anorexia	1
**Capecitabine:** Dermatological condition: rash	1
**Carfilzomib:** Hematologic: anaemia	1
**Ceritinib:** Gastrointestinal: diarrhoea	1
**Cladribine**: Pain	1
**Decitabine:** Renal impairment	1
**Doxorubicin:** Hypersensitivity: allergy	1
**Eribulin:** Neurological: neuropathy	1
**Erdafitinib:** Hematologic: thrombocytopenia	1
**Gilteritinib:** Cardiac impairment: QT elongation	1
**Ivosidenib:** Hematologic: differentiation syndrome	1
**Lapatinib:** Liver impairment	1
**Lorlatinib:** Gastrointestinal Immune-mediated: colitis	1
**Luthethium:** Renal impairment	1
**Nelarabine:** Neurological: myelopathy	1
**Olaratumab:** Neurological	1
**Osimertinib:** Hematologic: neutropenia	1
**Polatuzumab:** Renal impairment	1
**Radium dichloride:** Hematologic: anaemia	1
**Ribociclib:** Hematologic: febrile neutropenia	1
**Rucaparib:** Liver impairment	1
**Selpercatinib:** Renal impairment	1
**Siltuximab:** Liver impairment	1
**Temozolomide:** Renal impairment	1
**Topotecan:** Hematologic: neutropenia, thrombocytopenia, anaemia	1
**Vemurafenib:** Dermatological condition: rash	1

AE: adverse events; n=number of patients

Table S7. Overall Survival (OS) and Event-free Survival (EFS) for the five most frequent tumour types in the study of Hematological cancer and solid tumour.

Cancer types	n	Median OS, months (95% CI)	% 12 months OS (95% CI)	% 36 months OS (95% CI)	Median EFS, months (95% CI)	% 12 months EFS (95% CI)	% 36 months EFS (95% CI)
**Hematological cancer**							
Non-Hodgkin lymphoma	199	37.8 (21.2-63.8)	64% (57-71)	52% (46-60)	4.2 (3.3-5)	30% (24-37)	20% (15-27)
Acute myeloid leukaemia	86	10.0 (7.2-17.1)	45% (36-57)	24% (16-36)	2.4 (1.8-3.8)	15% (9-25)	6% (2-14)
Myelodysplastic/myeloproliferative syndrome	79	65.2 (33.2-84.6)	78% (70-88)	59% (48-71)	11.3 (5.9-22.4)	47% (37-59)	33% (24-46)
Hodgkin's lymphoma	57	35.8 (24.7-NR)	80% (70-91)	47% (35-63)	3.5 (2.4-4.9)	21% (13-35)	16% (9-29)
Multiple myeloma	47	24.6 (13.3-39.6)	64% (51-79)	39% (27-56)	4.4 (2.8-5.8)	26% (16-42)	11% (5-26)
**Solid tumour**							
Thoracic	361	15.9 (11.9-17.5)	55% (50-60)	30% (26-36)	4.2 (3.3-5)	30% (24-37)	20% (15-27)
Genitourinary	211	11.2 (8.8-15.2)	49% (43-57)	17% (12-23)	2.4 (1.8-3.8)	15% (9-25)	6% (2-14)
Breast	227	33.5 (26-41.6)	78% (72-83)	47% (40-54)	11.3 (5.9-22.4)	47% (37-59)	33% (24-46)
Skin	137	31.4 (20.8-47.1)	68% (60-76)	48% (39-58)	3.5 (2.4-4.9)	21% (13-35)	16% (9-29)
Gynaecological	133	29.9 (22.9-NR)	76% (69-84)	41% (30-55)	4.4 (2.8-5.8)	26% (16-42)	11% (5-26)

OS: overall survival; EFS: event-free survival; CI: confidence Interval; NR: non-reached

Table S8. Overall Survival (OS) and Event-free survival (EFS) for Hematologic malignancies subtypes (globally and by disease context).

Tumour subtype	Disease context	N	Median Overall Survival in months (95% CI)	Median Event Free-Survival in months (95% CI)
Acute lymphoblastic leukaemia	I/D	8	20.2 (8.6-NR)	1.5 (1.3-NR)
	R/R	32	7.3 (5.5-13.1)	2.1 (1.7-3.9)
	GLOBAL	40	8.8 (5.98 - 46.08)	1.9 (1.62 - 3.7)
Acute myeloid leukaemia	I/D	20	8.8 (6.4-23.8)	2.9 (1.7-9.9)
	R/R	69	11.1 (7.2-15.7)	2.5 (1.8-4.4)
	GLOBAL	89	9.8 (7.3 - 13.8)	2.5 (1.8 - 3.9)
Amyloidosis	R/R	16	69.6 (34.9-NR)	40.7 (6.6-NR)
Autoimmune thrombocytopenic purpura	I/D	8	96.5 (21.5-NR)	19.4 (7.4-NR)
	R/R	11	29.6 (16.2-NR)	20.4 (6.9-NR)
	GLOBAL	19	49.5 (24.9- NR)	19.44 (6.9 - NR)
Chronic lymphatic leukaemia	I/D	11	32.7 (19.4-NR)	22.4 (2.5-NR)
	R/R	31	26.9 (21.6-71.7)	11.8 (6.2-26.9)
	GLOBAL	42	31.2 (21.8 - 53.6)	11.8 (6.2 - 26.9)
Chronic myeloid leukemia	R/R	43	72.9 (35.7-NR)	30.9 (9.7-NR)
Diffuse large b-cell lymphoma	GLOBAL	36	6.5 (3.4 - 37.6)	2.2 (1.4 - 3.9)
Follicular lymphoma	R/R	30	63.7 (42.5-NR)	4.7 (4.2-19.9)
Hodgkin's lymphoma	I/D	1	NR (NR-NR)	NR (NR-NR)
	R/R	54	35.8 (26.6-NR)	3.3 (2.2-5.3)
	GLOBAL	55	35.8 (26.6 - NR)	3.5 (2.41 - 4.9)
Lymphoma-cell T	I/D	19	NR (NR-NR)	19.2 (5.1-NR)
	R/R	22	19.1 (6.4-NR)	2.1 (1.4-4.8)
	GLOBAL	41	55.4 (21.2 - NR)	4.5 (2.8 - 6.8)
Lymphoma-Diffuse large B-cell lymphoma	R/R	36	6.5 (3.4-37.6)	2.23 (1.4-3.9)
Mantle lymphoma	I/D	8	54.5 (18.9-NR)	4.8 (2.8-NR)
	R/R	23	9.2 (5.7-NR)	3.3 (0.9-6.9)
	GLOBAL	31	14.1 (7.8 - NR)	3.5 (1.5 - 6.1)
Marginal zone lymphoma	I/D	4	NR (NR-NR)	NR (NR-NR)
	R/R	9	36.3 (22-NR)	36.3 (24-NR)
	GLOBAL	13	37.8 (36.3 - NR)	36.3 (36.3 - NR)
Medullary aplasia	I/D	3	NR (NR-NR)	12.3 (4.2-NR)
	R/R	14	29.1 (16.1-NR)	4.8 (1.9-NR)
	GLOBAL	17	NR (18.05 - NR)	5.8 (1.9 - NR)
Multiple myeloma	I/D	11	65.9 (26.6-NR)	17.4 (5-NR)
	R/R	36	13.4 (8.3-34)	3 (2.3-5.4)
	GLOBAL	47	24.6 (13.3 - 39.6)	4.4 (2.8 - 5.8)
Myelodysplastic syndrome	R/R	33	24.0 (17-73.6)	5.06 (3.3-11.7)
Myeloproliferative syndrome - essential thrombocythemia	R/R	12	68.2 (68.2-NR)	57.6 (57.6-NR)
Myeloproliferative syndrome - myelofibrosis	I/D	9	53.7 (28.1-NR)	23.5 (9.1-NR)
	R/R	3	72 (65.3-NR)	11.3 (4.9-NR)

Tumour subtype	Disease context	N	Median Overall Survival in months (95% CI)	Median Event Free-Survival in months (95% CI)
	GLOBAL	12	65.2 (53.7 - NR)	21.6 (9.1 - NR)
Myeloproliferative syndrome - polycythemia vera	R/R	13	NR (NR-NR)	NR (NR-NR)
Non-Hodgkin's lymphoma – others	I/D	1	NR (NR-NR)	NR (NR-NR)
	R/R	18	6 (2.5-NR)	1.4 (0.6-7.8)
	GLOBAL	19	6.6 (2.64 - NR)	1.8 (0.7 - NR)
Other Hematologic tumours	I/D	11	NR (NR-NR)	7.7 (5.1-NR)
	R/R	61	32 (9.3-NR)	3.4 (1.9-6.9)
	GLOBAL	72	32 (14.4 - NR)	4.9 (2.8- 7.7)

I/D: induction, debut; R/R: refractory/relapsed; NR: non-reached, n=number of patients.

*For Hematological malignancies s with requests for both stages of disease, an additional row is added for the sum of both stages and its survival results (global).

Table S9. Overall Survival (OS) and Event-free survival (EFS) for solid tumour subtypes (globally and by disease context).

Tumour subtype	Disease context	N	Median Overall Survival in months (95% CI)	Median Event Free-Survival in months (95% CI)
Bliary tract	E	1	NR (NR-NR)	NR (NR-NR)
	A/M	1	NR (NR-NR)	NR (NR-NR)
	GLOBAL	2	15.3 (7.27 - NR)	2.7 (1.39 - NR)
Bladder	A/M	32	7.1 (3.7-11.8)	2.9 (1.4-6.5)
Breast	E	61	NR (NR-NR)	NR (NR-NR)
	A/M	162	22.6 (18.7-29)	6.3 (5.3-8.1)
	GLOBAL	223	34.9 (27.5 - 43.3)	7.7 (6.2 - 10)
Carcinoma of unknown origin	A/M	3	4 (2.7-NR)	1.8 (1.8-NR)
Central Nervous System	A/M	34	30.6 (9.1-NR)	6.2 (4.2-20)
Cervix	A/M	4	7.2 (3.3-NR)	3.8 (0.9-NR)
Colorectal	A/M	33	5.7 (4.1-8.6)	1.8 (1.1-3.7)
Endometrium	A/M	11	10.2 (1.6-NR)	5.6 (0.3-NR)
Gastric	E	2	NR (NR-NR)	4.2 (4.2-NR)
	A/M	6	10.5 (7.5-NR)	4.9 (2.1-NR)
	GLOBAL	8	19.9 (8.7 - NR)	5.11 (3.8 - NR)
Head and neck	A/M	31	9.6 (6.5-19.4)	3.0 (1.4-5.1)
Head and neck - thyroid	A/M	26	13.7 (4.4-23.3)	4.5 (2-13.4)
Kidney	A/M	59	10.4 (5.7-22.7)	3.7 (2.5-7.9)
Liver	A/M	16	9.4 (6-NR)	2.8 (1.9-10.9)
Melanoma	E	38	NR (NR-NR)	NR (NR-NR)
	A/M	37	17.4 (10.5-42)	4.1 (2.4-11.9)
	GLOBAL	75	38.0 (28.0 - NR)	11.47 (6.1 - 30.7)
Melanoma-targeted therapy	E	16	NR (NR-NR)	19.8 (9.3-NR)
	A/M	27	9 (6.6-22)	6.2 (4.6-10.6)
	GLOBAL	43	21.9 (13.5 - NR)	8.6 (5.7 - 19.8)
Mesothelioma	A/M	1	3.0 (NR-NR)	1.6 (NR-NR)

Tumour subtype	Disease context	N	Median Overall Survival in months (95% CI)	Median Event Free-Survival in months (95% CI)
Neuroendocrine tumour	A/M	45	21.3 (12.8-40.9)	8.5 (4.9-16.1)
Non-melanoma - Merkel cell carcinoma	A/M	10	59.7 (7.1-NR)	9.0 (2-NR)
Non-melanoma -squamous cell carcinoma	A/M	9	4.7 (2.6-NR)	1.4 (0.6-NR)
Non-small cell lung cancer - targeted therapy	A/M	148	20.1 (15.7-25.9)	10.1 (7.6-14.1)
Non-small cell lung cancer -not targeted therapy	E	28	42.1 (23.1-NR)	16.8 (6.2-NR)
	A/M	166	11.7 (10.2-16.6)	5.5 (3.9-6.6)
	GLOBAL	194	14.8 (11.1 - 18.3)	5.8 (4.2 - 7.2)
Oesophagus	E	1	NR (NR-NR)	NR (NR-NR)
	A/M	1	NR (NR-NR)	NR (NR-NR)
	GLOBAL	2	4.3 (4.0 - NR)	1.0 (0.5 - NR)
Ovarian nottargeted therapy	E	5	64.7 (34.3-NR)	4.7 (4.5-NR)
	A/M	21	13.1 (8.6-NR)	5.1 (3.6-8.5)
	GLOBAL	26	14.2 (11.1 - 64.7)	4.9 (3.7 - 7.2)
Ovarian-targeted therapy	A/M	92	34.0 (29.9-NR)	15.9 (8.8-NR)
Pancreas	E	4	29.8 (24.5-NR)	2.3 (1.4-NR)
	A/M	17	9.8 (6-29.4)	3.9 (1.4-13.5)
	GLOBAL	21	13.5 (7.3 - 29.8)	3.7 (1.4 - 13.5)
Prostate	A/M	120	13.9 (11-20.9)	5.1 (4.3-7.2)
Sarcoma	E	1	NR (NR-NR)	NR (NR-NR)
	A/M	82	16.6 (11.1-21.7)	3.3 (2.5-5.3)
	GLOBAL	83	16.6 (11.1 - 21.7)	3.6 (2.5 - 5.8)
Small-cell lung	A/M	10	3.7 (2.3-NR)	0.6 (0.2-NR)
Thymic carcinoma	A/M	4	13.6 (3.6-NR)	12.8 (2.6-NR)

A/M: advanced/metastatic; E: Early stage= (neo)adjuvant or localized stage

*For tumours with requests for both stages of disease, an additional row is added for the sum of both stages and its survival results (global).

Table S10. Survival according to the most frequent drug subclassifications in the study for Hematological cancer and solid tumour.

	n	Median Overall Survival in months (95% CI)	Median Event Free-Survival in months (95% CI)
**HEMATOLOGICAL CANCER**			
**Monoclonal antibody (iv)**			
ANTICD20	126	69.3 (56.9-NR)	6.7 (4.5-19.4)
ANTICD30	70	55.4 (33.2-NR)	4.5 (3.4-6.8)
**Targeted therapy (or)**			
IK-BCR/ABL	56	72.9 (46.1-NR)	16.5 (5.8-40.8)
IK-JAK	46	65.2 (25-NR)	19.6 (4.9-NR)
IK-BTK	36	26.9 (16.3-NR)	15.6 (6.9-36.3)
**SOLID TUMOUR**			
**Monoclonal antibody (iv)**			
ANTIPD-L1/PD-1	346	15.4 (11-6-18.6)	5.1 (3.7-6.5)
ANTIHER2	77	36.2 (30.1-59)	9 (6-17.6)
ANTIVEGF	34	16.6 (13.1-34.3)	7.8 (4.4-11.6)
**Targeted therapy (or)**			
IK-VEGF	122	9.8 (8.4-14.9)	3.9 (2.8-5.6)
IK-EFGR	99	16.2 (11.3-23.3)	9.5 (7.4-14.1)
I-PARP	104	34 (26.1-NR)	11 (7.9-26.1)
IK-CDK	67	29 (24.1-44.6)	10.2 (7.1-12.8)
IK-ALK	57	32.1 (16.2-58)	8.1 (5.5-22.6)
IK-BRAF	46	21.4 (14.1-NR)	8.4 (5.7-18.8)
IK-mTOR	27	24.2 (12.8-64.1)	6 (3.7-13.1)

NR: not reached; IK = Kinase Inhibitor; I: inhibitor; JAK: Global Janus kinase; BK:Bruton Kinase; CDK: cyclin-dependent kinases; ALK: anaplastic lymphoma kinase; mTor: Mammalian target of rapamycin; PD-L1/PD-1:Programmed death ligand L1/1; VEGF: Vascular Endothelial Growth Factor; EGFR: Epidermal Growth Factor Receptor; PARP: Poly Adenosine Diphosphate (ADP)-Ribose Polymerase; HER2: Human Epidermal Growth factor receptor 2;

Table S11. Frequency of drugs according to subclassifications for hematological cancer within monoclonal antibody and targeted therapy categories.

	Drug requested	n (%)
**Monoclonal antibody (iv)**		
ANTICD20 (n = 126)	Rituximab	114 (90.48%)
	Obinutuzumab	12 (9.52%)
ANTICD30 (n = 70)	Brentuximab vedotin	70 (100%)
**Targeted therapy (or)**		
IK-BCR/ABL (n = 56)	Ponatinib	16 (28.57%)
	Bosutinib	16 (28.57%)
	Dasatinib	10 (17.86%)
	Asciminib	9 (16.07%)
	Imatinib	4 (7.14%)
	Nilotinib	1 (1.79%)
IK-BTK (n = 82)	Ibrutinib	36 (100%)
IK-JAK (n = 46)	Ruxolitinib	46 (100%)

IK = Kinase Inhibitor; I: inhibitor; JAK: Global Janus kinase; BK:Bruton Kinase;

Table S12. Frequency of drugs according to subclassifications for solid tumour within monoclonal antibody and targeted therapy categories.

	Drug requested	n (%)
**Monoclonal antibody (iv)**		
ANTIHER2 (n = 77)	Trastuzumab emtansine	39 (50.65%)
	Pertuzumab + trastuzumab	22 (28.57%)
	Trastuzumab	15 (19.48%)
	Trastuzumab deruxtecan	1 (1.3%)
ANTIPD-L1/PD-1 (n = 346)	Nivolumab	142 (41.04%)
	Pembrolizumab	128 (36.99%)
	Atezolizumab	35 (10.12%)
	Durvalumab	28 (8.09%)
	Avelumab	5 (1.45%)
	Cemiplimab	7 (2.02%)
	Dostarlimab	1 (0.29%)
ANTIVEGF (n = 34)	Bevacizumab	34 (100%)
**Targeted therapy (or)**		
IK-ALK (n = 57)	Alectinib	21 (36.84%)
	Crizotinib	20 (35.09%)
	Brigatinib	9 (15.79%)
	Ceritinib	7 (12.28%)
IK-BRAF (n = 46)	Dabrafenib	41 (89.13%)
	Vemurafenib	3 (6.52%)
	Binimetinib + encorafenib	2 (4.35%)
IK-CDK (n = 67)	Palbociclib	50 (74.63%)
	Abemaciclib	11 (16.42%)
	Ribociclib	6 (8.96%)
IK-EGFR (n = 99)	Osimertinib	60 (60.61%)
	Afatinib	14 (14.14%)
	Lorlatinib	10 (10.1%)
	Sorafenib	9 (9.09%)
	Rociletinib	2 (2.02%)
	Dacomitinib	1 (1.01%)
	Erlotinib	1 (1.01%)
	Gefitinib	1 (1.01%)
	Mobocertinib	1 (1.01%)
IK-mTOR (n = 27)	Everolimus	27 (100%)
IK-VEGF (n = 122)	Regorafenib	28 (22.95%)
	Nintedanib	17 (13.93%)
	Pazopanib	18 (14.75%)
	Sunitinib	15 (12.3%)
	Axitinib	16 (13.11%)
	Lenvatinib	11 (9.02%)

	Cabozantinib<br>Vandetanib<br>Tivozanib	9 (7.38%)<br>7 (5.74%)<br>1 (0.82%)
IPARP (n = 104)	Olaparib<br>Niraparib<br>Rucaparib	57 (54.81%)<br>45 (43.27%)<br>2 (1.92%)

IK = Kinase Inhibitor; I: inhibitor; JAK: Global Janus kinase; BK: Bruton Kinase; CDK: cyclin-dependent kinases; ALK: anaplastic lymphoma kinase; mTor: Mammalian target of rapamycin; PD-L1/PD-1: Programmed death ligand L1/1; VEGF: Vascular Endothelial Growth Factor; EGFR: Epidermal Growth Factor Receptor; PARP: Poly Adenosine Diphosphate (ADP)-Ribose Polymerase; HER2: Human Epidermal Growth factor receptor 2.

Table S13. Overall survival and Event-free survival for oncology drugs requested more than ten times, for an indication eventually approved by the European Medicines Agency (EMA) and results obtained for the drug in pivotal trials.

Drug	Approved uses	Study drug results			Published drug results				ESMO - MCBS
		N= 887	mOS (m) (95% CI)	m EFS (m) (95% CI)	mOS (m) drug	mOS control (m)	mPFS (m) drug	mPFS control (m)	
**Abiraterone + prednisone**	2nd line castration-refractory after docetaxel	55	18.1 (13.1-22.6)	6.2 (5.3-10)	15.8	11.2	5.6	3.6	4
**Afatinib**	As monotherapy for the treatment of EGFR TKI-naïve adult patients with locally advanced or metastatic NSCLC with activating EGFR mutation(s)	13	11.5 (5.7-NR)	4.3 (1.9-NR)	23	23	11.1	6.9	4
**Alectinib**	As monotherapy is indicated for the treatment of adult patients with ALK-positive advanced NSCLC previously treated with crizotinib	21	50.3 (23.6-NR)	28.1 (13.9-NR)	NR	NR	10.9	1.4	4
**Alpelisib**	In combination with fulvestrant treatment of postmenopausal women, and men, with hormone receptor-positive, HER2-negative, locally advanced or metastatic breast cancer with a PIK3CA mutation after disease progression following ET as monotherapy	11	19.4 (13.7-NR) *previous iCDK4/6	5.5 (0.9-NR)	39.3	31.4	11	5.7	2
**Atezolizumab**	Patients with locally advanced or metastatic urothelial carcinoma who are considered cisplatin ineligible, and whose tumours have a PD-L1 expression ≥5%	21	3.7 (1.9-8.3)	1.4 (0-4.5)	15.2	13.3	NA	4.1	3
**Axitinib**	Treatment of adult patients with advanced renal cell carcinoma after failure of prior treatment with sunitinib or cytokine	16	13.3 (6.4-29.8)	4.5 (2.5-13.6)	20.1	19.2	6.7	4.7	4
**Bevacizumab**	In combination with paclitaxel, topotecan, or pegylated liposomal doxorubicin for platinum-resistant recurrent epithelial ovarian	10	12.1 (6.3-NR)	5.2 (3.6-NR)	16.6	13.3	6.7	3.4	4
**Cabazitaxel**	Metastatic castration-resistant prostate cancer after docetaxel and AST inhibitor	18	6.6 (4.3-12.1)	3.3 (2.5-5.1)	13.6	11	8	3.7	3
**Dabrafenib+/- trametinib**	Unresectable or metastatic melanoma with a BRAF V600 mutation	19	14.1 (8.8-NR)	8.3 (5.7-NR)	25.1	18.7	11	8.8	4
	adjuvant treatment of Stage III melanoma with a BRAF V600 mutation, following complete resection	16	NR (NR-NR)	19.8 (9.3-NR)	NR	16.6	NA	NA	A
**Durvalumab**	Locally advanced, unresectable NSCLC in adults whose tumours express PD-L1 on ≥1% of tumour cells and whose disease has not progressed following platinum-based ChT	28	42.15 (23.1-NR)	16.8 (6.2-NR)	47.5	29.1	17.2	5.6	4
**Enzalutamide**	2nd line castration-refractory after docetaxel	29	23.0 (12.3-51.7)	8.4 (2.7-22.8)	18.4	13.6	8.3	2.9	4
**Everolimus**	Metastatic breast cancer after failure of aromatase inhibitor	12	26.4 (10.5-NR)	5.5 (2.7-NR)	31	26	10.6	4.1	2
	Unresectable or metastatic, well-differentiated neuroendocrine tumours of gastrointestinal or lung origin in adults with progressive disease	11	21.7 (15.2-NR)	13.1 (3.9-NR)	44.02	37.68	11	3.9	3

Drug	Approved uses	Study drug results			Published drug results				ESMO - MCBS
		N= 887	mOS (m) (95% CI)	m EFS (m) (95% CI)	mOS (m) drug	mOS control (m)	mPFS (m) drug	mPFS control (m)	
**FU + liposomal irinotecan**	Metastatic adenocarcinoma of the pancreas progressed following gemcitabine-based therapy	10	7.7 (5.8-NR)	3.8 (0.9-NR)	6.1	4.2	3.1	1.5	3
**Imatinib**	Kit (CD 117) positive unresectable and/or metastatic malignant GIST	13	40.1 (17.4-NR)	7.6 (2.8-NR)	NR	-	NA	-	NA
**Ipilimumab**	Second-line treatment in advanced (unresectable or metastatic) melanoma	10	22.9 (11.5-NR)	10.2 (2.4-NR)	10.1	6.4	NA	NA	4
**Lenvatinib**	Locally advanced or metastatic, differentiated thyroid carcinoma refractory to radioactive iodine	11	4.4 (3.9-NR)	3.9 (3-NR)	4.77	2.07	18.3	3.6	2
**Lorlatinib**	ALK+ advanced NSCLC after TKI therapy	10	16.5 (10.2-NR)	14.4 (5.6-NR)	NA	-	6.9	6.9	4
**Lutethium**	Gastroenteropancreatic neuroendocrine tumours (GEP-NETs).	12	NR (30.7-NR)	16.1 (8.5-NR)	61.2	-	28.5	-	4
**Nintedanib**	With docetaxel for locally advanced, metastatic or locally recurrent NSCLC after first line ChT.	17	9.5 (6.9-20)	2.1 (1.9-5.6)	12.6	10.3	4.2	2.8	NA
**Niraparib**	Platinum-sensitive relapsed high-grade serous epithelial ovarian cancer who are in response to platinum-based ChT	45	26.1 (21.4-NR)	4.8 (3.7-9.3)	40.9 (mut)-31	38.1 (mut)-34.8	9.3-21	3. 9(mut)-5.5	3
**Nivolumab**	Recurrent or metastatic SCCHN	20	9.6 (6.5-28.6)	2.5 (1.4-4.2)	9.2	6.01	3.5	2.8	5
	Advanced (unresectable or metastatic) melanoma	30	NR (NR-NR)	31.4 (10.6-NR)	16.8	10.8	5.1	2.2	4-A
	2nd line after platinum-based therapy advanced non-squamous-cell NSCLC stratified for PD-L1	57	9.9 (5.8-13.9)	3.3 (2.4-6.5)	12.2	9.4	NA	NA	5
	Advanced renal cell carcinoma after prior therapy in adults	21	5.6 (2.6-24.7)	2.3 (1.4-12.2)	25.0	19.6	4.6	4.4	5
**Olaparib**	Maintenance treatment for platinum-sensitive relapsed high-grade epithelial ovarian cancer who are in response to platinum-based chemotherapy	45	41.3 (34-NR)	34.1 (34-NR)	34.9	30.2	11.2	4.3	2
**Osimertinib**	Previously untreated EGFR mutated (exon 19 deletion or L858R)	36	17.8 (14.6-NR)	13.9 (8.3-NR)	38.6	31.8	18.9	10.2	4
	2nd line for EGFR mutated NSCLC after TKI with new T790M mutation	23	14.1 (8-41.7)	14.1 (7.4-27.4)	26.8	22.5	10.1	4.4	4
**Palbociclib**	HER2-negative, HR+ advanced or metastatic breast cancer previously treated with endocrine therapy	35	33.8 (24.1-NR)	11.3 (8.1-13.8)	34.9	28	9.5	4.6	4
**Palbociclib**	First-line postmenopausal, ER-positive, HER2-negative locally advanced metastatic breast cancer	14	41.6 (29-NR)	11.7 (4.4-NR)	53.9	51.2	24.8	14.5	3

Drug	Approved uses	Study drug results			Published drug results				ESMO-MCBS
		N= 887	mOS (m) (95% CI)	m EFS (m) (95% CI)	mOS (m) drug	mOS control (m)	mPFS (m) drug	mPFS control (m)	
**Pazopanib**	Previously treated non-GIST metastatic soft tissue sarcoma	14	5.9 (4.9-NR)	1.8 (1-6.4)	12.6	10.7	4.6	1.6	3
**Pembrolizumab**	Advanced (unresectable or metastatic) melanoma	22	42.0 (4.6-NR)	11.0 (2.2-NR)	13.4	11	2.9	2.7	3
	2nd line after platinum-based therapy or TKI (for EGFR/ALK Mutated) advanced NSCLC>1% tumour cell PD-L1 expression	25	16.6 (10.1-NR)	7.3 (2.8-23.9)	10.4	8.4	3.9	4.1	5
**Pembrolizumab + carboplatin + pemetrexed**	First line metastatic squamous non-small cell lung cancer	52	16.6 (11.9-NR)	8.6 (6-15.7)	15.9	11.3	6.4	4.8	4
**Regorafenib**	Advanced GIST	14	18.9 (13.8-49.2)	10.5 (6.7-38.4)	NR	NR	4.8	0.9	3
**Trastuzumab emtansine**	HER2-positive locally advanced or metastatic breast cancer previously treated with trastuzumab and a taxane	19	30.1 (14.7-56.7)	6.2 (3-17.6)	30.9	25.1	9.6	6.4	4
**Trastuzumab emtansine**	Adjuvant treatment of patients with HER2-positive early breast cancer who have residual invasive disease in the breast and/or lymph nodes after neoadjuvant taxane-based and HER2-targeted therapy	18	NR (NR-NR)	NR (NR-NR)	NR	NR	14.2	9.2	A

mOS: median overall survival; mEFS: median event-free survival; mPFS: median progression-free survival; m: months; NA: non-available; NR: non-reached; Mut: BRCA mutated; TKI: tirosin kinase inhibitor; ER: estrogen receptor; ALK: anaplastic lymphoma kinase; NSCLC: non-small cell lung cancer; ChT: chemotherapy; SCCHN: Squamous cell carcinoma of head and neck; endocrine therapy; FU: fluorouracil; GEP-NETs: Gastroenteropancreatic neuroendocrine tumours; EGFR: Epidermal Growth Factor Receptor; CDK: cyclin-dependent kinases; AST: Androgen suppression treatment.

Table S14: Overall survival and Event-free survival for Hematology drugs requested more than ten times, for an indication eventually approved by the European Medicines Agency (EMA) and results obtained for the drug in pivotal trials.

Drug	Approved uses	Study drug results			Published drug results			
		N= 225	mOS (m) (95% CI)	mEFS (m) (95% CI)	mOS (m)	mOS (m) control	mPFS (m)	mPFS (m) control
**Bosutinib**	Previously treated chronic myeloid leukaemia (CML)	16	NA (72.9-NA)	41.1 (7.3-NA)	83.5	-	NA	-
**Brentuximab vedotin**	With cyclophosphamide, doxorubicin, and prednisone for systemic anaplastic large cell lymphoma previously untreated	12	55.4 (NA -NA)	16.5 (5.1-NA)	NR	NR	55.6	54.1
	Relapsed Hodgkin lymphoma	27	35.9 (26.6-NA)	3.5 (2.2-7.7)	40.5	-	NA	-
	CD30+ cutaneous T-cell lymphoma after at least one prior treatment	15	NA (22.6 -NA)	5.2 (1.7-NA)	NA	NA	16.7	3.5
**Daratumumab**	Relapsed multiple myeloma, as monotherapy	14	14.25 (7-45.3)	2.8 (2.3-12)	17.5	NA	NA	NA
**Decitabine**	De novo or secondary AML, who are not candidates for conventional induction chemotherapy	10	6.6 (2.5-NA)	2.8 (1.7-NA)	7.7	5	NA	NA
**Eltrombopag**	Acquired severe aplastic anaemia refractory to prior immunosuppressive therapy or heavily pre-treated	11	29.1 (16.1-NA)	3.8 (1.9-NA)	NA	NA	NA	NA
**Ibrutinib**	CLL in previously treated patients	13	26.9 (17-NA)	14.6 (6.6-NA)	58.8	NA	21.1	NA
**Inotuzumab ozogamicin**	Relapsed or refractory CD22-positive B cell precursor acute lymphoblastic leukaemia	11	8.8 (7-NA)	2.1 (1.7-NA)	7.7	6.2	NA	NA
**Lenalidomide**	Transfusion-dependent anaemia due to low- or intermediate-1-risk myelodysplastic syndromes	13	55.4 (29-NA)	9.1 (5.1-NA)	NA	NA	NA	NA
**Nivolumab**	Relapsed or refractory classical Hodgkin lymphoma after ASCT and treatment with brentuximab vedotin.	12	31.9 (19.6-NA)	6.3 (4.9-NA)	NR	90 (82. 94)	NA	NA
**Obinutuzumab-Bendamustine**	Follicular lymphoma who did not respond or who progressed during or up to 6 months after treatment with rituximab	10	63.7 (58.1-NA)	18 (4.7-NA)	NR	NR	NR	13.8
**Ponatinib**	CML resistant or intolerant to dasatinib or nilotinib, imatinib is not clinically appropriate; or who have the T315I mutation	12	NA (16.9-NA)	28.6 (8.9-NA)	NR	-	NA	-
**Ruxolitinib**	Graft versus host disease with inadequate response to corticosteroids or other systemic therapies	23	6.7 (2.6-NA)	3 (1.3-NA)	NA	-	NA	-
	PolycytHemia vera who are resistant to or intolerant of hydroxyurea.	10	NA (NA-NA)	NA (NA-NA)	NA	-	NA	-
**Venetoclax + azacytidine**	In combination with a hypomethylating agent, for the treatment of newly diagnosed AML	14	89.0 (2.2-NA)	2.4 (1.7-NA)	14,7	9,6	9.8	7

N=number of requests; mOS: median overall survival: mEFS: median event-free survival; mPFS: median progression-free survival; m: months; NA: non-available, NR: non-reached; CML: Chronic myeloid leukaemia; ASCT: autologous stem cell transplant; CLL: chronic lymphocytic leukaemia; AML: acute myeloid leukemia.
